# Survivin and Aurora Kinase A control cell fate decisions during mitosis

**DOI:** 10.1002/1878-0261.70141

**Published:** 2025-10-12

**Authors:** Hana Abdelkabir, Shalitha Wickrama Arachchige, Sally P. Wheatley

**Affiliations:** ^1^ School of Life Sciences University of Nottingham UK

**Keywords:** aurora kinase, chromosomal passenger complex (CPC), mitosis, mitotic slippage, spindle assembly checkpoint (SAC), survivin

## Abstract

The spindle assembly checkpoint (SAC) delays the metaphase‐to‐anaphase transition. Aurora kinase A (AURKA) inactivation has been shown to cause premature exit from mitosis in the presence of an unsatisfied SAC. We report for the first time that centromeric AURKA interacts with survivin during prometaphase. Notably, depleting or inhibiting AURKA activity at this stage causes mislocalisation of the CPC and BubR1, which compromises the SAC and can lead to mitotic slippage. Furthermore, we show that AURKA binds directly to the BIR domain of survivin at a position distinct from AURKB and indirectly to it via its C terminus. We find the interaction peaks during prometaphase but persists into late mitosis. Importantly, we demonstrate that cells with high levels of survivin are particularly vulnerable to mitotic slippage induced by the AURKA inhibitor, MLN8237/ Alisertib. Alisertib enables both normal and transformed cells with high levels of survivin to activate the APC/C prematurely, as observed by the destruction of cyclin B and securin. Thus, a high expression of survivin can alter cell fate decisions at mitosis and lead to genetic instability, a key hallmark in cancer.

## Graphical abstract

Aurora A interacts with survivin during mitosis and regulates its centromeric role. Loss of Aurora A activity mislocalises survivin, the CPC and BubR1, leading to disruption of the spindle checkpoint and triggering premature mitotic exit, which we refer to as ‘mitotic slippage’. Elevated survivin levels compromise the effectiveness of Aurora A inhibitors, highlighting a potential limitation for targeted cancer therapies.

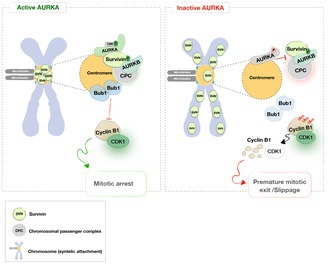

**Abbreviations**
APC/C: anaphase promoting complex/cyclosome
AURKA: aurora kinase A
AURKB: aurora kinase B
aurora kinase C: aurora‐C
CPC: chromosomal passenger complex
KT: kinetochore
MT: microtubule
SAC: spindle assembly checkpoint

## 1 Introduction

In humans, the aurora family of serine/threonine kinases has 3 members: aurora kinase A (AURKA), aurora kinase B (AURKB), and aurora kinase C (AURKC). Both AURKA and AURKB are expressed in all mitotic cells and are 57% identical, while aurora C is principally expressed in the testes [1, 2, 3], although it has been shown to complement AURKB loss in some systems [2, 4, 5]. AURKA and AURKB also share similar phosphorylation consensus sequences, their substrate specificity thought to be determined by their distinct activators and different locations within the cell [3]. In early mitosis, AURKA is predominantly located at the centrosomes and the base of the microtubules emanating from each pole [6, 7]. Together with its principal activator, TPX2 [8, 9, 10], centrosomal AURKA plays a vital role in constructing the bipolar mitotic spindle and is activated in its kinase domain by polo‐like kinase (plk1) [11]. However, this is not the only pool of AURKA present in early mitosis: recently, it has been discovered that some AURKA is present at the plus ends of kinetochore microtubules and the centromeres [12, 13, 14]. Previously thought to be a domicile exclusive to AURKB of this kinase family [15, 16], it is now clear that this pool of AURKA influences chromosomal passenger complex (CPC) targeting to the centromere [14].

The CPC is a tetrameric mitotic complex composed of AURKB, borealin, INCENP and survivin ([17]; reviewed in [18]). Located at the centromeres in prometaphase, the CPC communicates to the spindle assembly checkpoint (SAC) to ensure chromosomes align correctly at the centre of the cell before segregating at anaphase. The CPC is targeted to the centromere by its smallest component, survivin, which recognises and binds to histone H3 once it has been phosphorylated at threonine 3 (Thr3) by the mitotic kinase, haspin [19, 20, 21, 22]. A second mechanism involving Bub1 phosphorylated histone H2A, borealin, and shogushin is also involved in ensuring correct CPC targeting [23, 24].

During prometaphase, maloriented chromosomes that have not attached to the spindle correctly activate the spindle assembly checkpoint (SAC), the main function of which is to delay the transition from metaphase to anaphase by inhibiting the E3‐ubiquitin ligase known as the Anaphase Promoting Complex/Cyclosome (APC/C). Multiple mitotic kinases, such as Mps1, Bub1 and BubR1, are involved in SAC signalling to keep the APC inactive until all the kinetochores are attached to the MTs [25, 26, 27]. Biochemically, the APC is regulated by the mitotic checkpoint complex (MCC), a diffusible inhibitor established by three checkpoint proteins, BUB3, BUBR1, and MAD2 [28, 29], which interact with cell division cycle protein 20 (CDC20), an activator of the APC, and keep it in an inactive form until all kinetochores are attached [30]. Once a bipolar attachment between the spindle microtubules and the sister kinetochores at the sides of each chromosome is achieved, the MCC disassembles, the anaphase inhibitory proteins diffuse, and the levels of Mad2, Bub1 and BubR1 plummet [28, 29]. APC activation leads to cyclin B degradation and thus inactivation of cyclin‐dependent kinase 1 (CDK1) [31, 32, 33], as well as securin destruction, which promotes chromosome separation via degrading the cohesin ties between the sister chromatids via the release of separase [34].

If the cell is unable to correct its maloriented chromosomes and the SAC persists, cells should eventually be eliminated by apoptosis. However, cells endure different fates, fluctuating between mitotic slippage and death, and the occurrence of one or the other depends on the cell type [35, 36] and which network threshold is reached first [37]. Mitotic slippage, which describes cells that escaped mitotic arrest, is governed by APC/C inactivation and a drop in cyclin B1 levels [38, 39], due to APC activation [40]. This undesirable outcome yields a single polyploid cell with 4 N DNA content, rather than two daughter cells with 2 N DNA, and thus, it propagates genetic instability within the organism [38]. On the other hand, apoptosis is governed by the activation of pro‐apoptotic caspases and inhibition of the anti‐apoptotic proteins Bcl‐2 and Bcl‐xL by active CDK1, as well as CDK1‐directed trafficking of pro‐apoptotic proteins Bax and Bak to the mitochondria [41].

Various studies have demonstrated the importance of survivin in regulating the SAC through mediating the activity of its CPC partner, AURKB, which has been linked to SAC recruitment during early mitosis [42]. AURKB activity is required for the recruitment of the SAC proteins, BubR1 and MAD2, to the kinetochore, and its inhibition eventually results in a failure in detecting any microtubule‐kinetochore (MT‐KT) mis‐attachment [42, 43]. AURKB phosphorylates Hec1, a member of the CDC80 complex, and promotes its interaction with Mps1 to specify its localisation at the kinetochores [44]. Mps1, which is a vital kinase that works upstream of SAC, phosphorylates the outer kinetochore protein KNL1, which consequently mediates the recruitment of BubR1 and MAD2 at the unattached kinetochores [45, 46].

Although SAC regulation has been linked to AURKB activity, various lines of evidence have shown that AURKA inactivation causes premature exit from mitosis in the presence of unsatisfied SAC [12, 47]. Like AURKB, AURKA regulates KT‐MT attachment by phosphorylating Hec1 [48, 49] to enable adjustment of KT‐MT attachment errors during prometaphase [50]. Various lines of evidence indicate that AURKA shares similar centromeric substrates and interactors with AURKB during mitosis [51, 52]. In fact, a study by Fu et al. [53] showed that a replacement of a single amino acid allowed mutant AURKA‐G198N to interact with AURKB mitotic partners such as survivin and INCENP [53]. However, despite the evidence provided indicating the possibility of collaboration between survivin and AURKA in regulating mitosis, until now there has been no proof of their association. In this paper, we show for the first time that AURKA binds to survivin during mitosis and regulates its function and also provide evidence that centromeric AURKA and AURKB are not functionally redundant. Importantly, we report that prometaphase cells that express aberrantly high levels of survivin are able to side‐step AURKA‐targeted oncotherapeutics and exacerbate, rather than prevent, genome instability due to mitotic slippage.

## 2 Materials and methods

All materials used were from Sigma‐Aldrich unless otherwise stated.

### 2.1 Mammalian cell culture, mitotic synchronisation and drug treatments

HeLa, MRC‐5 and U2OS cells (ATCC) and the derived stable cell lines of each expressing GFP or survivin‐GFP were cultured as monolayers at 37 °C with 5% CO_2_ in a humid environment using high glucose Dulbecco's modified Eagle's medium (D6429), supplemented with 10% foetal calf serum (FCS) (F7524) and 1% Antibiotic Antimycotic Solution (Thermo Fisher Scientific, Waltham, MA, USA). 1 mm G418 Sulphate (Thermofisher Scientific) was added to the medium used for cells expressing GFP/survivin‐GFP maintenance. Cells authenticated within the past 3 years were checked every 3 months for mycoplasma contamination using PCR and only used if mycoplasma‐free. Cells were synchronised in prometaphase for 6 h, or overnight for 12–14 h, using the Eg5 inhibitor, Dimethylenastron (2 μm, DMA, 5261; TOCRIS Biosciences, Abington, UK), as described in Wheatley [54]. To obtain cells in metaphase, DMA‐arrested (Eg5i) cells were released into the proteasomal inhibitor 20 μm MG132 (Z‐Leu‐Leu‐Leu‐al) and harvested 3 h later. MLN8237, known clinically as “Alisertib” (Selleckem Chemicals LLS, Houston, TX, USA), was used at 0.25 μm or 1 μm to inhibit AURKA. Barasertib‐HQPA (AZD1128) (AstraZeneca Pharmaceuticals, Cambridge, UK) was used at 0.5 μm AZD to inhibit AURKB. All drugs were prepared in DMSO, filter sterilised and stored at −20 °C, then diluted directly into medium.

### 2.2 Short interfering RNA (siRNA) transfection

Cells were seeded at 60% confluency 24 h prior to the day of transfection. Both control siRNA (Cat #43908490) and AURKA siRNA (Cat #AM51331) consist of a proprietary sequence provided by Invitrogen (Thermo Fisher Scientific) and used at a final concentration of 40 nm. Transfection was performed using Lipofectamine™ 2000 transfection reagent (Thermo Fisher Scientific) diluted in antibiotic‐free Opti‐MEM™ I Reduced Serum Medium (Gibco, Invitrogen) as recommended by the manufacturer.

### 2.3 Immunofluorescence imaging

0.1 × 10^6^ cells were seeded onto sterilised coverslips coated with 10% (v/v) poly‐L‐lysine (150–300 kDa), placed in a 12‐well plate, then incubated at 37 °C for 24 h before treatment. For immunostaining, coverslips were washed (3× 5 min, PBS), then incubated for 5 min in 4% formaldehyde (v/v) (Electron Microscopy Sciences), rewashed (3× 5 min PBS), then permeabilised in 0.15% (v/v) Triton‐x‐100 for 2 min and washed again. The fixed and permeabilised cells were then blocked in PBS‐based 1% BSA containing 0.1% sodium azide (Merck), then incubated with primary antibodies diluted in the BSA‐blocking buffer for either 1 h at RT or overnight at 4 °C. They were then washed and incubated for 1 h in the dark with the relevant secondary antibodies diluted in the BSA‐blocking buffer containing 1 μg·mL^−1^ DAPI stain (4′,6‐diamidino‐2‐phenylindole; Thermo Fisher Scientific). After a final washing step, the coverslips were dried and mounted on glass slides using Mowiol mounting medium. Images were captured with a Zeiss 200 M microscope using a 63× (1.40 NA, oil immersion) objective and ZenBlue software and processed in Fiji (1.0). Thresholding was applied across channels using predefined scales. Intensity analysis required manual region selection, maintaining consistent regions of interest (ROIs). Co‐localisation was evaluated using the RGB line plot profiler (Fiji 1.0).

### 2.4 Immunoblotting

Cells were harvested by scraping in cold PBS, and whole cell extracts (WCE) were prepared in RIPA buffer (20 mm Tris/HCl pH 7.4, 137 mm NaCl, 1% NP‐40, 1% deoxycholic acid, 0.1% SDS, 10% glycerol), containing phosphatase inhibitors [2 mm β‐glycerophosphate, 1 μg·mL^−1^ CLAP (chymostatin, leupeptin, antipain, pepstatin A), 100 μm PMSF, 20 mm Na_3_VO_4_, 2 mm MgCl_2_, and 4 units·mL^−1^ DNase (Quanta Biosciences, Beverly, MA, USA)]. To lyse, cells were incubated on ice for 30 min, then sonicated twice for 10 s at 25 Hz (Vibro‐Cell Ultrasonic sonicator). Protein concentrations were determined using the Bradford assay (Bradford 1976). Using standard 12 or 15% SDS/PAGE gels, proteins were separated and then transferred to a 0.22 μm nitrocellulose membrane (Cytivia, Marlborough, MA, USA). Membranes were either blocked for 1 h with 5% non‐fat dry milk (MARVEL) or 5% BSA in TBST (150 mm NaCl, 50 mm Tris, 0.1% Tween‐20, pH 7.4), then incubated overnight at 4 °C with primary antibodies. HRP‐conjugated secondary antibodies (DAKO) were then applied at room temperature for 1 h, and bands were visualised using Enhanced Chemiluminescence (ECL) (Cytivia) reagent, followed by exposure to photographic film (Kodak, Rochester, NY, USA).

### 2.5 Antibodies used in the study

Primary antibodies against the following proteins were used for immunoblotting (IB) and diluted in blocking buffer 5% (v/v) milk TBST or 5% (v/v) BSA TBST or used for immunofluorescence (IF) and diluted in blocking buffer 1% BSA/0.1% azide in PBS. Anti‐GST HRP‐conjugated (RPN1236V; 1 : 10000) from Cytiva, anti‐GFP (1 : 1000 for IB only), and Borealin (1 : 200 for IB, 1 : 100 for IB) (in‐house). Anti‐Aurora A (D3E4Q; 1 : 200 for IF, 1 : 1000 for IB), Survivin (71G4B7; 1 : 100 for IF, 1 : 1000 for IB), Survivin (6E4; 1 : 200 for IF), INCENP (P240; 1 : 100 for IF, 1 : 1000 for IB) and phospho‐Aurora (A/B/C) (D13A11; 1 : 1000 for IF; Cell Signalling Technology CST, Danvers, MA, USA). Aurora B (ab2254; 1 : 100 for IF, 1 : 1000 for IB) and B512 (Tubulin) (T5168; 1 : 2000 for IF, 1 : 1000 for IB) from Sigma. CENP‐C (ab50974; 1 : 500 for IF only), Non‐Muscle Myosin IIB (ab264266; 1 : 200 for IF), b‐Actin (2D4H5; 1 : 500 for IB) and Securin (EPR3240; 1 : 1000 for IB) from Abcam. BubR1 (1 : 500 for IF, 1 : 1000 for IB) was a gift from Prof. S. S. Taylor, University of Manchester, UK. Cyclin B1 (554 177; 1 : 100 for IF, 1 : 500 for IB) and Lamin B1 (1 : 100 for IF) were from BD Bioscience, and Histone H3S10ph pAb (39 254; 1 : 5000 for IB) was from Active Motive. Secondary antibodies used for IB: HRP‐anti‐mouse (1 : 750), HRP‐anti‐rabbit and HRP‐anti‐sheep (1 : 1000) diluted in blocking buffer 5% (v/v) milk TBST or 5% (v/v) BSA TBST as recommended for the primary (DAKO). For (IF): FITC‐anti‐mouse, Texas red anti‐mouse, FITC‐anti‐rabbit, Texas red anti‐rabbit, Texas red anti‐sheep (1 : 500) diluted in blocking buffer 1% BSA/0.1% azide in PBS (Vectorlabs, Newark, CA, USA).

### 2.6 GFP trap

Stable cell lines expressing GFP or GFP‐tagged survivin at 80% confluency were harvested by scraping into ice‐cold PBS. WCE was prepared by 30 min incubation on ice in lysis buffer (10 mm Tris/HCl pH 7.5, 150 mm NaCl, 0.5 mm EDTA, 0.5% NP‐40), supplemented with CLAP (1 μg·mL^−1^), PMSF (100 μm), β‐glycerophosphatase (2 mm), DNaseI (0.2 U·mL^−1^), and MgCl_2_ (2 mm). Samples were then sonicated 3 times (10 s, 25 Hz, on ice), then centrifuged to remove debris. GFP‐Trap beads (Proteintech, Rosemount, IL, USA) were vortexed, then 25 μL of slurry beads were used for each reaction. The beads were washed with an ice‐cold dilution buffer consisting of Tris/HCl pH 7.5, 150 mm NaCl, and 0.5 mm EDTA 3× by centrifuging the beads at 2500 × ***g*** for 2 min at 4 °C. 500 μL of cell lysate was added to each reaction and incubated for 2 min on a rotating wheel at 4 °C. The beads then were washed (4 × in dilution buffer) by centrifuging the samples at 2500 × ***g*** for 2 min at 4 °C. The beads were resuspended in 50 μL 2× SDS sample buffer. The samples were incubated at 95 °C for 5 min and stored at −20 °C until analysis by immunoblotting.

### 2.7 Recombinant protein expression, GST‐pulldowns

For expression, *E. coli* BL21 cells were transformed with pGEX4T1 vectors and grown from single colonies in LB‐ampicillin broth to OD_600_ nm of 0.6. Protein expression was induced with 0.5 mm isopropyl β‐D‐1‐thiogalactopyranoside (IPTG) (16 h, 20 °C, 240 ***g***). Bacterial cells were harvested by centrifugation (4000 ***g***, 15 min), then lysed in ice‐cold TBS containing 1 nm β‐mercaptoethanol, 1 μg·mL^−1^ CLAP (chymostatin, leupeptin, antipain and pepstatin A), deoxyribonuclease DNase25 (100 mg), and 2 mm MgCl_2_ (BDH). After lysis, samples were sonicated 12 × (10 s, 50 Hz on ice), using a Vibra‐Cell™ ultrasonic sonicator before centrifugation at 4000 × ***g*** (4 °C for 30 min). To purify the GST‐tagged proteins, the bacterial supernatant was incubated with Glutathione‐Sepharose 4B beads (GE Healthcare Life Science; 500 μL of slurry beads per 2.5 mg of protein) for 1 h at 4 °C with rotation. The beads were then washed 3× with distilled water, followed by 3× 15 min washes with TBST (20 mm Tris/HCl, pH 8.0, 150 mm NaCl, 0.1% Tween 20) and a final overnight wash with TBS. Proteins were eluted from the beads using 1 mL of 10 mm reduced glutathione, prepared in 50 mm Tris/HCl pH 8.0 buffer (30 min, 4 °C, with rotation). The beads were then washed (3× with elution buffer) and then dialysed using dialysis tubing cellulose membrane overnight at 4 °C in 50 mm Tris/HCl pH 8.0, 150 mm NaCl. Prewashed (3× distilled water, 3× PBS) GST‐beads were then incubated with the purified GST‐tagged fusion proteins (1 h, 4 °C with rotation), then washed twice with ice‐cold TBS. For the interaction assay, the beads with bound protein were incubated with whole asynchronous or mitotically synchronised cell extracts (see preparation details in the immunoblotting section) or IVT‐produced protein on a rotating wheel for 2 h at 4 °C. For IVT, a TNT‐T7 coupled reticulocyte kit was used as directed by the manufacturer (Promega). Proteins of interest were expressed from mammalian expression vectors (see Table S1). After incubation with cell extract/IVT protein, the beads were centrifuged at 500 × ***g*** and washed 3×. The first and second washes were in 50 mm Tris/HCl (pH 8.0, containing 150 mm NaCl with 1% NP‐40), and the third wash was with 50 mM Tris/HCl pH 8.0, with 1% NP‐40 without NaCl. Finally, the beads were boiled in 2× SDS sample buffer (95 °C, 5 min) and stored at 20 °C until analysis by immunoblotting.

### 2.8 Molecular cloning

All cloning reagents were from NEB unless otherwise stated.

Cloning strategy for survivin constructs, EcoR1 – Xho1 in pGEX4T1, with GFP C‐terminal to survivin after removal of the stop codon where appropriate. All variants have been used previously, except the BIR domain, 20–90, and DD70‐71AA. The pGEX4T1 BIR domain was synthesised by PCR using standard methods: 25 ng of wild‐type (WT) survivin plasmid as a template, 0.02 UI·μL^−1^ Q5 hot start polymerase, 0.5 μm primers fwd 5′‐GGCGAATTCTCTACATTCAAGAAC‐3′; reverse 5′‐CAACTCGAGTCACTTGACAGAAAG‐3′, 200 μm deoxynucleotide triphosphates (dNTPs), and the PCR program used was 95 °C (3 min) initial denaturation, 39 cycles (95 °C, 30 s denaturing; 60 °C, 30 s annealing; 72 °C, 30 s polymerisation), followed by 72 °C, 5 min, final elongation. The PCR product was then cut and pasted into the pGEX4T1 vector using EcoR1 and Xho1 restriction sites and T4 DNA ligase using standard methods. All constructs were verified by sequencing prior to use.

### 2.9 Statistics

Data sets were exported to Excel and analysed in graphpad prism 10.2. A two‐tailed, unpaired Student's *t*‐test was used to compare the means of two independent groups. One‐way ANOVA was used for data sets containing more than two samples. For more complicated methodologies involving repeated measurements, a two‐way ANOVA was conducted. ANOVA analysis was followed by either a *post hoc* Tukey's multiple comparisons test or a Dunnett's multiple comparisons test.

## 3 Results

### 3.1 AURKA interacts with survivin during mitosis

**Fig. 1:**
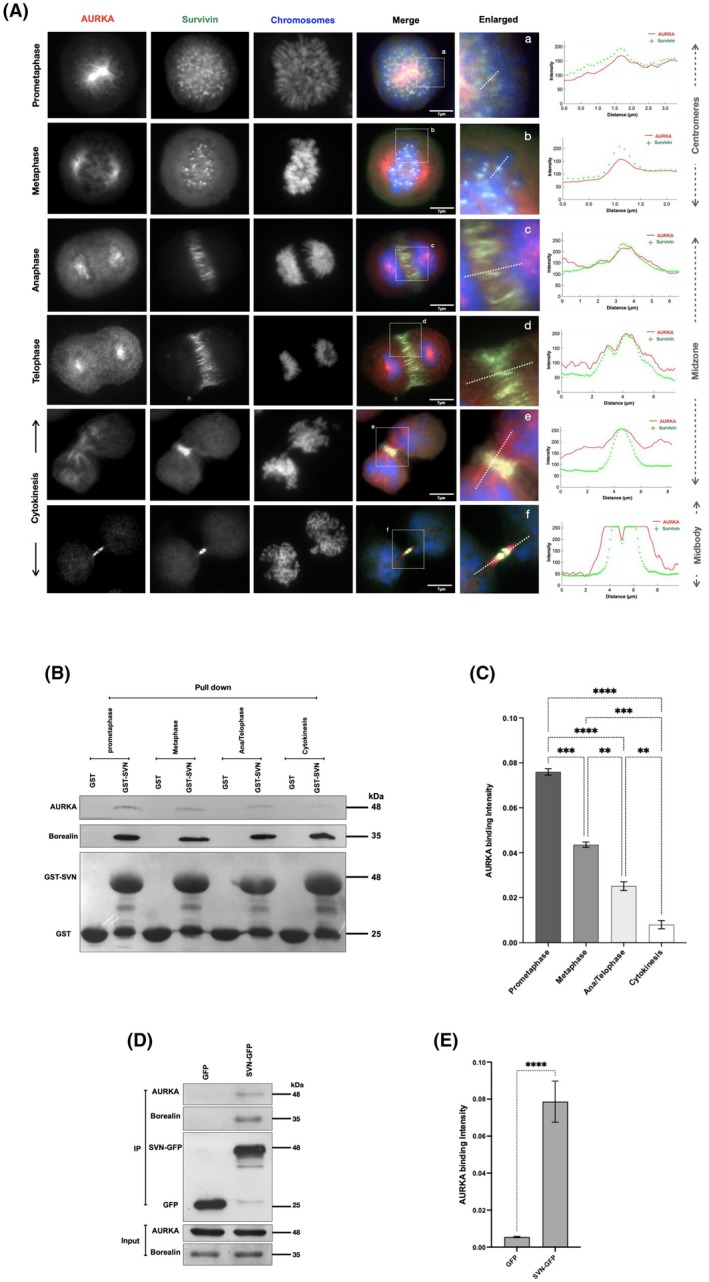
A subpopulation of AURKA associates with survivin throughout mitosis. (A) Mitotic HeLa cells fixed and immunostained to detect the localisation of both AURKA (red) and survivin (green). DAPI (blue) was used to stain the chromosomes. Fluorescence intensity profiles were measured along the white dashed lines in the enlarged images. Scale bar: 7 μm. (B) Immunoblot of a GST pull‐down assay from synchronous mitotic HeLa cells demonstrates GST‐survivin (GST‐SVN) and its affinity towards endogenous AURKA compared to the negative control (GST). (C) Graph illustrates an ordinary one‐way ANOVA analysis of the variation in GST‐survivin binding affinity towards AURKA across different mitotic stages, normalised to the GST/GST‐SVN intensity. (D) Representative immunoblot of immunoprecipitates from stable HeLa cell lines expressing GFP (negative control) or survivin‐GFP (SVN‐GFP), pulled down using a GFP‐Trap and analysed by immunoblotting with antibodies against borealin (positive control), AURKA and GFP. (E) Graph demonstrates an ordinary one‐way ANOVA analysis of the intensity of AURKA co‐immunoprecipitated with survivin‐GFP, normalised to the GFP/SVN‐GFP intensity. All blots and microscopy images shown are representative of three independent repeats. Quantitative data are presented as means ± SD (*n* = 3). *P* value (ns = non‐significant, **P < 0.01, ***P < 0.001, ****P < 0.0001).

To investigate whether survivin associates with AURKA during mitosis, first we carried out dual localisation by immunostaining HeLa cells with anti‐survivin and anti‐AURKA antibodies. During prometaphase and metaphase, the majority of AURKA was located at the centrosomes and base of the mitotic spindle; however, another population was simultaneously detected colocalising with survivin at the centromeres (Fig. 1A (panels a and b)). To highlight this, an intensity profile was plotted (see enlarged image), which shows AURKA peaks at the centromeres coinciding with those of survivin. At anaphase, survivin relocates from the centromeres to the central midzone (Fig. 1A (panels c)), where it persists until abscission. During these late stages of mitosis, while the majority of AURKA remains centrosomal, it gradually becomes more microtubule‐associated as cytokinesis progresses, and a fraction of AURKA continues to colocalise with survivin until the two nascent daughter cells form (Fig. 1A (panels c–f)).

As we witnessed co‐localisation between survivin and AURKA throughout mitosis, we hypothesised that they interact at some or all of these stages. To test this, HeLa cells were synchronised using DMA and either held in the arrested state (prometaphase; 100%) or released into drug‐free medium and harvested 1 h later (anaphase–telophase; 54%), 2 h later (cytokinesis; 88%), or released into 20 μm MG132 for 3 h (metaphase; 75%); see Fig. S1A for the experimental plan. Cells were then collected onto poly‐lysine‐coated coverslips and fixed and immunostained for α‐tubulin to confirm their mitotic status phenotypically (Fig. S1B). The percentage of cells in the desired stage is indicated in the brackets above; see Fig. S1C for the full profile. Synchronised cells were then lysed and immunoblotted with anti‐cyclin B1 antibodies as a mitotic marker and tubulin as a loading control. As shown in Fig. S1D, the levels of cyclin B1 were comparable in the prometaphase‐arrested and MG132‐treated (metaphase) populations, indicating that they were arrested in early mitosis, while in the released samples, a noticeable loss of cyclin B1 was apparent 1 h post‐release, which was completely absent 2 h post‐release, indicating mitotic exit had been achieved in these populations (Fig. S1D,E).

Interaction between survivin and AURKA was then assessed using a GST pull‐down experiment with the mitotically synchronised cell lysates. As shown in Fig. 1B, we found that the interaction between AURKA and survivin was in its strongest state in prometaphase and declined but persisted in anaphase/telophase, reducing almost to zero during cytokinesis (Fig. 1B,C). *In cellulo* interaction with endogenous AURKA was also confirmed using a GFP trap in combination with lysates prepared from asynchronous GFP and survivin‐GFP‐expressing HeLa cells and immunoblotting with anti‐AURKA antibodies (Fig. 1D,E). Together these results indicate that survivin interacts with AURKA during early mitosis, and their interaction is at its highest during prometaphase, at the centromeres where they both colocalise.

### 3.2 Survivin binds directly to AURKA via its BIR domain

**Fig. 2:**
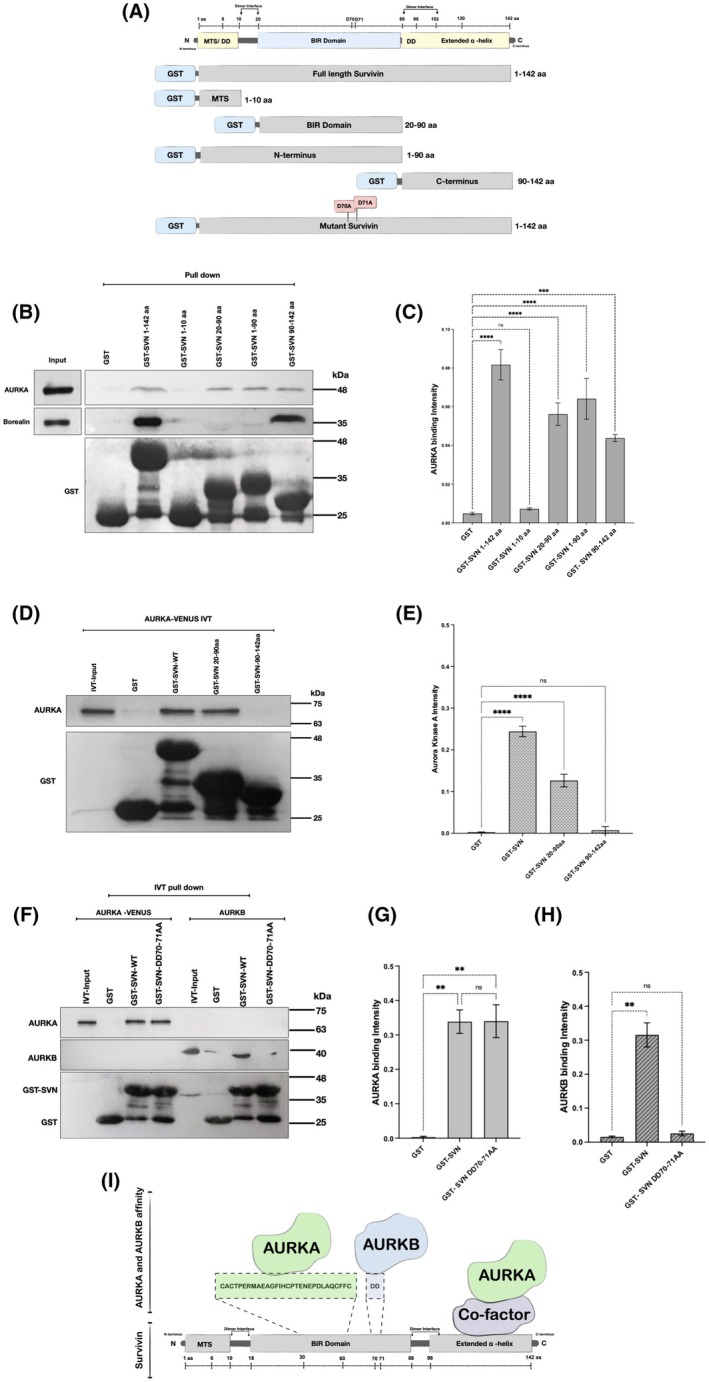
Survivin interacts directly with AURKA via its BIR domain and indirectly via its C‐terminus. To map the interaction site(s) between survivin and AURKA, a GST pull‐down assay was conducted using asynchronous HeLa cell extracts. (A) Cartoon schematic presentation of survivin structure and the different truncations and mutations used in the pull‐down presented as follows: full‐length survivin GST‐SVN‐1‐142aa, GST‐SVN‐1‐10aa, GST‐SVN‐20‐90aa, GST‐SVN‐1‐190aa, GST‐SVN‐90‐142aa, and the double point mutation GST‐SVN(DD70‐71AA). GST was used as a negative control. (B) Immunoblot of GST pull‐down assay demonstrates the different truncations of survivin and their affinity towards AURKA when incubated with asynchronous HeLa cell extract. Borealin was used as a positive control. (C) Graph demonstrates an ordinary one‐way ANOVA analysis of the intensity of AURKA pulled down by the different truncations of survivin compared to GST, normalised to the GST or GST‐survivin bands. (D) Immunoblot demonstrates *in vitro* TNT‐T7 coupled reticulocyte assay. Venus‐tagged AURKA was *in vitro* translated and incubated with purified GST‐SVN 1–142 aa (full‐length survivin) and its truncations GST‐SVN 20–90 aa (BIR domain) and GST‐SVN 90–142 aa (C‐terminus). (E) Graph demonstrates an ordinary one‐way ANOVA analysis of the intensity of the *in vitro* translated AURKA bound directly to survivin and its mutant truncations compared to GST, normalised to the GST or GST‐survivin bands. (F) Immunoblot represents the affinity of the *in vitro* translated Venus‐AURKA and AURKB towards GST recombinant survivin (wild type) and its mutant GST‐SVN (DD70,71AA). (G, H) Graphs demonstrate an ordinary one‐way ANOVA analysis of the intensity of the *in vitro* translated AURKA/AURKB, which bound directly to survivin and its mutant version compared to the negative control, GST only, normalised to the GST/GST‐survivin bands. (I) Schematic diagram summarises the different sites of interaction between AURKA and AURKB within survivin. AURKA interacts with survivin directly via the BIR domain (amino acids 30 to 60) and indirectly via its C‐terminus. AURKB binds directly to the BIR domain of survivin via its amino acids D70D71. All blots shown are representative of three independent repeats. Quantitative data are presented as means ± SD (*n* = 3). *P* value (ns = non‐significant, **P < 0.01, ***P < 0.001, ****P < 0.0001).

Having established that survivin and AURKA co‐localise and co‐immunoprecipitate *in cellulo*, we next used the GST pulldown assay using a variety of survivin truncations and point mutations (Fig. 2A for schema) to map the interaction site(s). When these experiments were carried out with whole cell HeLa extracts (WCE) as ‘prey’, immunoblotting revealed that both the BIR domain (20–90 aa) and the C terminus (90–142 aa) were able to bind to AURKA (Fig. 2B,C). However, when the pull‐down was performed using *in vitro* translated (IVT) AURKA‐Venus expressed from a mammalian expression vector to allow us to identify whether the interactions were direct, only the BIR domain segment of survivin bound to AURKA (Fig. 2D,E).

Next, as AURKB binds to survivin via amino acids D70 and D71 within the BIR domain [55], we tested the double mutant (D70A/D71A) to see whether the interaction site was common to both AURKs. Interestingly, while the interaction between survivin and AURKB was completely abolished by these substitutions, the interaction with AURKA was unaffected (Fig. 2F–H). Further mapping revealed the direct interaction to involve residues 30–60 in the BIR domain, but the precise amino acids remain to be identified (Fig. S2A–C). Four substitutions within this region were also tested: T34A/E and T48A/E, which are sites phosphorylated by CDK1 [56, 57] and CK2 [58], respectively, but neither the non‐phosphorylatable (T to A) mutants nor the phosphomimetics (T to E) prevented AURKA binding to survivin (Fig. S2D,E).

Collectively, these data show that there are two AURKA interaction sites within survivin: one indirect via the C terminus and one that is direct involving the BIR domain, but that is distinct from the AURKB site and not affected by phosphostatus at T34 or T48 and that lies within the region aa 30–60, as summarised in Fig. 2I.

### 3.3 Depleting AURKA causes survivin and CPC mislocalisation during mitosis

**Fig. 3:**
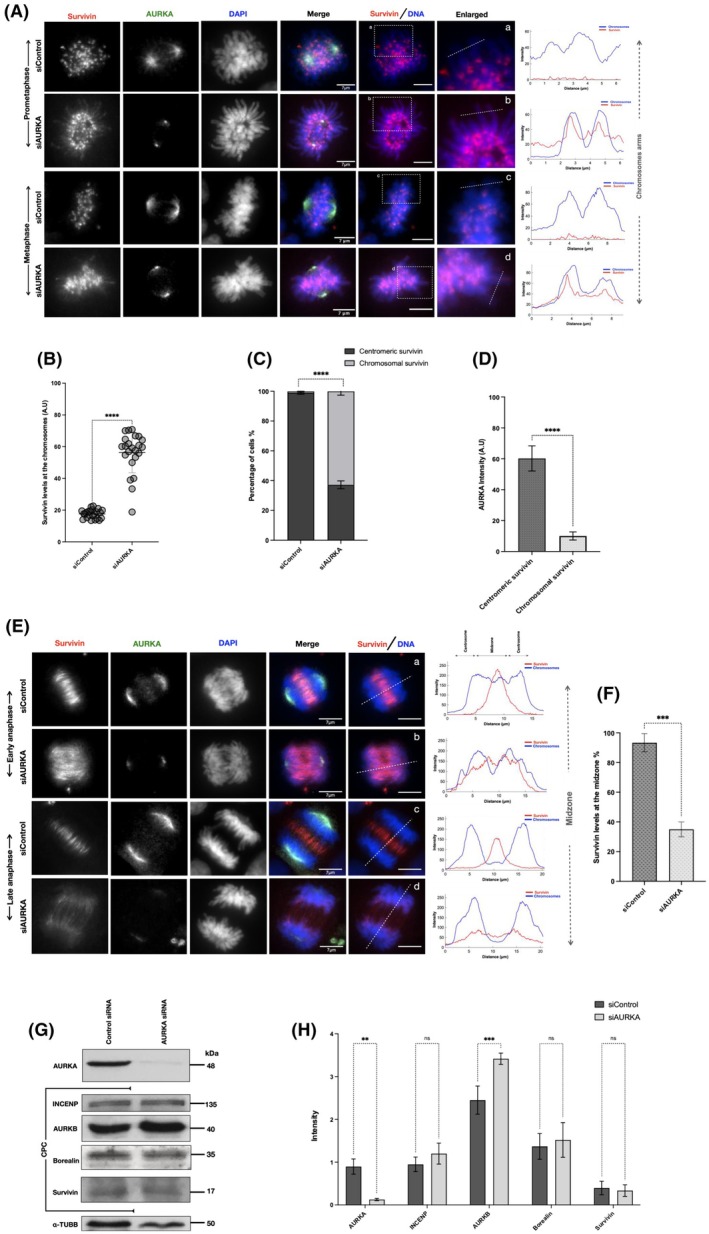
Depleting AURKA affects survivin localisation during early mitosis. (A) Prometaphase and metaphase HeLa cells were treated with either 40 nm scrambled siRNA (siControl) or 40 nm AURKA siRNA for 48 h, then fixed and immunostained to examine survivin localisation. Panels (a, b) represent prometaphase. Panel (c, d) represent metaphase. AURKA (green), survivin (red). Chromosomes are stained with DAPI (blue). Fluorescence intensity profiles were measured along the chromosome arms to examine the localisation of survivin. Scale bar: 7 μm. (B) Graph demonstrates a Student's *t*‐test analysis of the mean intensity of survivin at the chromosome arms of cells treated with 40 nm siAURKA for 48 h compared to the control, presented in arbitrary units (AU). (C) Graph demonstrates a two‐way ANOVA analysis of the differences in the number of cells with chromosomal survivin in cells treated with siAURKA compared to siControl, presented in percentage. Number of cells with correct localisation of survivin (centromeric) in siControl‐treated HeLa cells (*n*1 = 29) (*n*2 = 34) (*n*3 = 27) and in siAURKA‐treated HeLa cells (*n*1 = 8) (*n*2 = 7) (*n*3 = 8). Number of cells with mislocalised survivin (chromosomal) in siControl‐treated HeLa cells (*n*1 = 1) (*n*2 = 0) (*n*3 = 0) and in siAURKA‐treated HeLa cells (*n*1 = 15) (*n*2 = 12) (*n*3 = 12). (D) Graph demonstrates a Student's t‐test analysis of the differences in AURKA mean intensity, presented in arbitrary units (AU), in HeLa cells treated with siAURKA with mislocalised survivin (chromosomal) compared to HeLa cells with correctly localised survivin (centromeric). (E) HeLa cells treated with either 40 nm siControl or 40 nm siAURKA for 48 h were fixed and immunostained to examine survivin localisation in late mitosis (anaphase). Panels (a, b) represent cells in early anaphase. Panels (c, d) represent late anaphase. AURKA (green), survivin (red) and chromosomes (blue). Fluorescence intensity profiles were obtained to examine the localisation of survivin at the midzone. Scale bar: 7 μm. (F) Graph demonstrates a Student's t‐test analysis of the mean intensity of survivin at the midzone area, presented in percentage. (G) Western blot of HeLa cell lysate treated with either 40 nm siControl or 40 nm siAURKA for 48 h. The lysate was immunoblotted using antibodies against AURKA and the CPC members (INCENP, AURKB, borealin and survivin). α‐tubulin was used as a loading control. (H) Graph shows a two‐way ANOVA analysis of the efficiency of AURKA knockdown and its impact on the levels of survivin and the other members of the CPC. Band intensity was normalised to α‐tubulin. All blots and microscopy images shown are representative of three independent repeats. Quantitative data are presented as means ± SD (*n* = 3). *P* value (ns = non‐significant, **P < 0.01, ***P < 0.001, ****P < 0.0001).

To begin to investigate the functional relevance of this interaction, we first depleted AURKA using siRNA. As presented in Fig. S3A,B, via immunoblotting with anti‐AURKA and normalising band intensity to the tubulin loading control, we found that treating cells for 24 h with AURKA‐siRNA reduced expression of AURKA by 20%, while 48 h increased the efficacy of AURKA silencing by 75%; therefore, 48 h was used in all siRNA experiments. To determine whether AURKA depletion affected survivin localisation, we carried out immunostaining with anti‐survivin (red) and AURKA antibodies (green) on HeLa cells after treatment with either control siRNA (siControl) or AURKA‐specific siRNA (siAURKA); see Fig. 3A. To identify the mitotic stage of the cells, anti‐tubulin immunostaining was used, and the centromeres were visualised using anti‐CENP‐C antibodies (Fig. S4).

During early stages of mitosis (prometaphase and metaphase), in stark contrast to siControl cells, survivin was observed diffused along the chromosome arms in the siAURKA population rather than focused on the centromeres (Figs 3A and S4). To confirm these observations, a line intensity analysis was conducted using Fiji software to quantitate survivin localisation, showing coincidence of survivin with the chromosome arms (Fig. 3A (panels a–d),B) rather than with CENPC peaks (Fig. S4, panels e & f), in almost 63% of cells treated with siAURKA (Fig. 3C). Quantification of pixel intensity revealed that the majority of cells with chromosomal rather than centromere‐focused survivin were those with the lowest expression of AURKA (Fig. 3D), proving that these two parameters are correlated.

Consistent with AURKA being necessary for spindle construction, we also found a dramatic increase in multipolar spindles (63.8%) in the siAURKA population compared to siControl‐treated controls (5%), see Fig. S5A,B. Importantly, survivin mislocalisation was observed in all cells with lowered AURKA regardless of their spindle status (Fig. S5A,C); thus, we are confident that the survivin mislocalisation is not an indirect consequence of spindle malformation. From these observations, we conclude that AURKA depletion causes mislocalisation of survivin away from the centromeres and along the chromosome arms during early mitosis.

Interestingly, although only very few cells made it into metaphase and beyond as a consequence of AURKA inhibition, in those that did, less than 40% of the survivin population migrated to the central spindles, with the majority remaining chromosomal, as highlighted in the line profiles through the central midzone in the enlarged panels (Fig. 3E,F).

Since one of the most important functions of survivin is targeting the CPC to the centromeres during early mitosis, we next examined the localisation of the CPC members in these cells. Using the abnormal distribution of survivin as a surrogate indicator of AURKA depletion, we observed that AURKB (Fig. S6), as well as borealin and INCENP (Fig. S7), were misplaced similarly to survivin when AURKA was depleted. Finally, using immunoblotting, we asked whether the overall levels of the CPC members were affected by AURKA depletion. Interestingly, while borealin, INCENP and survivin levels remained constant, AURKB levels rose significantly in the absence of AURKA (Fig. 3G,H).

Collectively, these findings indicate that AURKA acts as a regulator of survivin localisation during both early and late mitosis and that maintaining normal expression levels of AURKA is essential for the correct localisation of survivin and the CPC during mitosis.

### 3.4 Inhibiting AURKA activity causes premature exit from mitosis when survivin is upregulated

Next, as it has been shown that AURKB activity is enhanced by survivin overexpression [59, 60], we asked whether survivin had a similar effect on AURKA activity. Therefore, the impact of overexpressing survivin on AURKA autophosphorylation and consequently on cells' resistance towards AURKA inhibitors during early mitosis was examined. To assess the level of survivin overexpression in our experimental model, survivin protein levels were quantified by western blotting and normalised to β‐actin. As shown in Fig. S8D,E, survivin‐GFP expression was approximately 23‐fold higher than endogenous survivin in asynchronous cells and around 10‐fold higher in mitotically arrested cells.

Different concentrations of MLN8237 (MLN), a selective inhibitor of AURKA, were tested to determine the optimal concentration required for achieving full inhibition of AURKA without affecting AURKB; see Fig. S8. To achieve this, HeLa cells were arrested in early mitosis using DMA, then released into MG132 for 45 min to ensure arrest in metaphase, the stage during which AURKA activation is documented to be at its highest [12], then treated with MLN (see Fig. S8A). Cells were then lysed, and the levels of p‐AURKA and p‐AURKB were detected using antibodies against pT228 and pT232, respectively, and cyclin B1 levels were used as a mitotic indicator. A noticeable increase in the activation levels of both AURKs was observed upon arrest; however, a full reduction of p‐AURKA without impacting p‐AURKB was achieved using 0.25 μm MLN; see Fig. S8B,C.

**Fig. 4:**
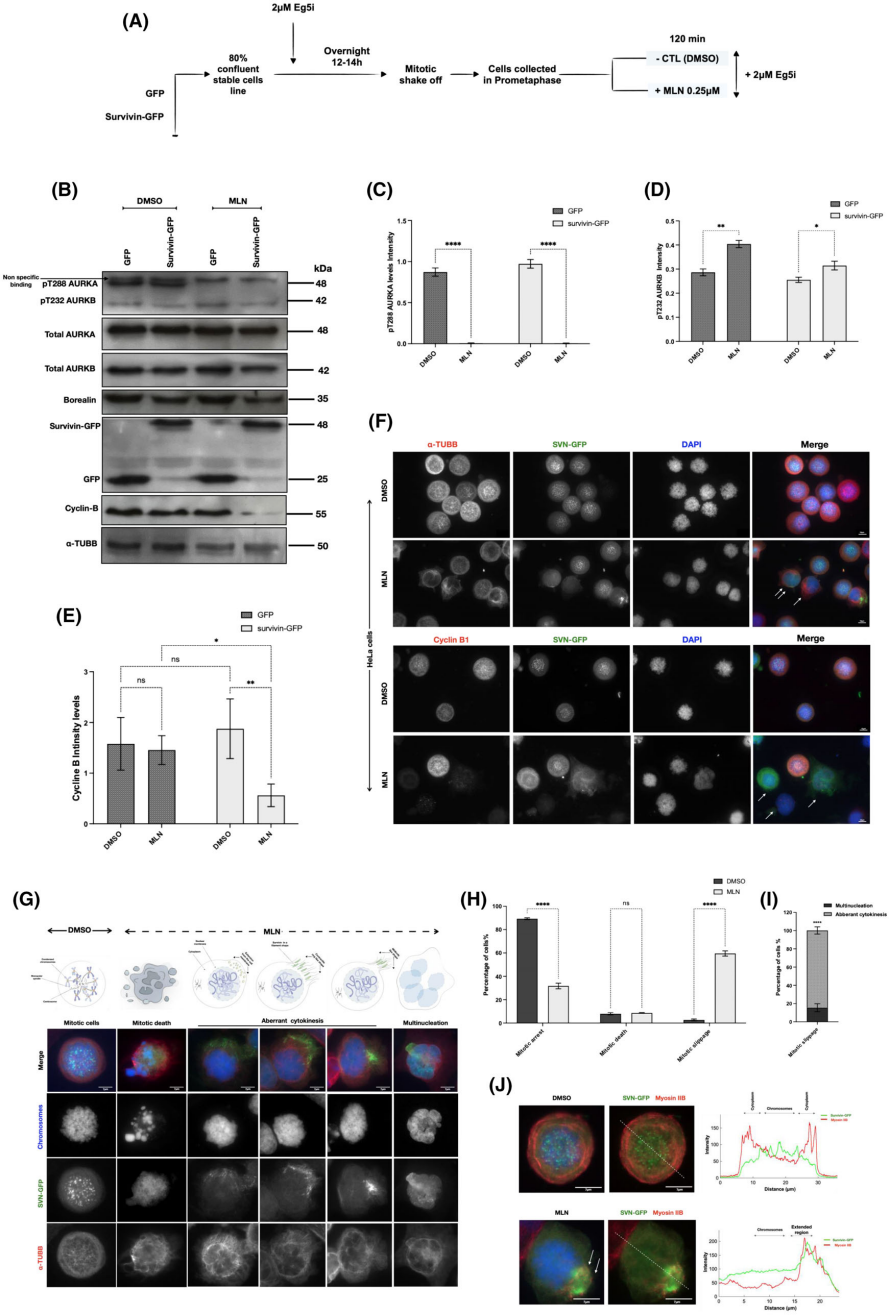
Inhibiting AURKA activity during prometaphase in survivin‐GFP‐expressing cells causes mitotic slippage. (A) Schematic demonstrating the method used for cell synchronisation and treatment. HeLa stable cell lines expressing GFP or survivin‐GFP were arrested with 2 μm Eg5 inhibitor overnight, then treated for 120 min with either DMSO (negative control) or 0.25 μm MLN8237 (MLN). (B) Immnoblot of mitotically arrested stable HeLa cell lines lysate expressing GFP or survivin‐GFP and treated with either DMSO (CTL) or 0.25 μm MLN. A black arrow indicates non‐specific bands. Immunoblotting against pThr288 AURKA and pThr232 AURKB was used to determine AURKA and AURKB activity, respectively. α‐tubulin was used as a loading control, and cyclin B1 as a mitotic marker. (C–E) Graphs demonstrate two‐way ANOVA analysis of the expression intensity levels of pThr288 AURKA, pThr232 AURKB and cyclin B, respectively, normalised to α‐tubulin (α‐TUBB), in DMSO and 0.25 μM MLN‐treated HeLa cells. (F) Images of HeLa cell line expressing survivin‐GFP fixed and immunostained for α‐tubulin or cyclin B1 (red). DAPI stain was used to mark the nucleus/chromosomes (blue) and survivin tagged to GFP in (green). White arrows indicate non‐mitotic cells. Scale bar: 10 μm. (G) Images of a stable HeLa cell line expressing survivin‐GFP demonstrating the different fates observed following 0.25 μm MLN treatment. Scale bar: 7 μm. (H) Graph demonstrates two‐way ANOVA analysis of the differences in the percentage of HeLa cells undergoing different cell fates upon 0.25 μm MLN treatment compared to DMSO. Number of mitotic cells in DMSO (*n*1 = 376) (*n*2 = 357) (*n*3 = 481), 0.25 μm MLN (*n*1 = 123) (*n*2 = 118) (*n*3 = 122). Number of mitotic deaths in DMSO‐treated cells (*n*1 = 30) (*n*2 = 32) (*n*3 = 38) and 0.25 μm MLN‐treated cells (*n*1 = 33) (*n*2 = 36) (*n*3 = 31). Number of mitotic slippage in DMSO (*n*1 = 15) (*n*2 = 9) (*n*3 = 10) and in 0.25 μm MLN (*n*1 = 234) (*n*2 = 246) (*n*3 = 204). (I) Graph demonstrates a *Student's t‐*test of the percentage of HeLa cells with aberrant cytokinesis compared to multinucleated following mitotic slippage induced by 0.25 μm MLN. Number of cells with aberrant cytokinesis following treatment with 0.25 μm MLN (*n*1 = 198) (*n*2 = 197) (*n*3 = 181) and multinucleated cells (*n*1 = 36) (*n*2 = 49) (*n*3 = 23). (J) Images illustrate the localisation of survivin and myosin IIB in a stable HeLa cell line expressing survivin‐GFP during premature mitotic exit after treatment with 0.25 μm MLN compared to DMSO. White arrows highlight the extended region in MLN‐treated cells undergoing mitotic slippage. Fluorescence intensity profiles were obtained along the white dashed lines shown in the images. Cells were immunostained for myosin IIB (red), chromosomes (blue), and survivin‐GFP (green). Scale bars: 7 μm. All results in this figure were obtained from HeLa cells stably expressing either GFP or survivin‐GFP following the experimental design shown in (A). All blots and microscopy images shown are representative of three independent repeats. Quantitative data are presented as means ± SD (*n* = 3). *P* value (ns = non‐significant, *P < 0.05, **P < 0.01, ***P < 0.001, ****P < 0.0001).

Next, to examine whether upregulating the levels of survivin impacts cell sensitivity towards the AURKA inhibitor MLN, HeLa stable cell lines expressing either GFP or survivin‐GFP were synchronised using an Eg5 inhibitor overnight. The mitotically arrested cells were treated with either control (DMSO) or 0.25 μm MLN for 120 min, as presented in Fig. 4A. Cells were then lysed and examined using immunoblotting; see Fig. 4B. The levels of p‐AURKA and p‐AURKB were detected in each blot to examine the efficiency of the treatment. We also probed for cyclin B1, with the intention that this would prove that the cells in each condition were indeed in mitosis.

Interestingly, as demonstrated in Fig. 4B,C, overexpressing survivin did not cause a noticeable increase in the phosphorylation levels of AURKA, nor did it increase cellular resistance to MLN treatment. However, AURKB phosphorylation levels were noticeably increased as a result of inhibiting the phosphorylation levels of AURKA, which suggests the possibility of a compensation mechanism between AURKs; see Fig. 4B–D. Surprisingly and notably, we found that while cyclin B was present at high levels in all DMSO‐treated cells and GFP‐MLN‐treated cells, they were reduced significantly in SVN‐GFP cells treated with MLN (Fig. 4E). Since a drop in the expression levels of cyclin B indicates a change in the mitotic status of the mitotically arrested cells, further investigations were required to examine the fate of cells overexpressing survivin when AURKA activity is inhibited. This immunofluorescence imaging was used alongside immunoblotting to examine the fate of SVN‐GFP (green) populations via staining with DAPI (blue) and immunostaining with either tubulin or cyclin B1 antibodies (red) (see Fig. 4F). In agreement with the immunoblotting data, we found that SVN‐GFP treated with DMSO remained in the prometaphase arrest with condensed chromosomes and clear punctate centromeric SVN‐GFP foci. In contrast, in the majority of SVN‐GFP cells treated with MLN, the chromosomes appeared decondensed, tubulin dispersed, cyclin B absent, and survivin either diffuse in the cell or accumulated to one side in filaments (Fig. 4F).

Through closely examining the status of the DNA in HeLa cells expressing survivin‐GFP, which escaped mitotic arrest following treatment with MLN, it was found that some of the cells underwent apoptosis (Fig. 4G (panel b)). However, the percentage of SVN‐GFP cells with this fate was low and consistent in both DMSO‐ and MLN‐treated populations, compared to the significant increase in cells that underwent mitotic slippage (60% in MLN‐treated cells versus 3% controls) (Fig. 4H).

Interestingly, around 15% of the cells that slipped mitosis became multinucleated (Fig. 4G (panel f),H). However, around 60% exhibited abnormalities in division, with survivin observed accumulated in filaments or in an extended region which simulated the midbody formed in late mitosis, which indicates a failed attempt to divide or aberrant cytokinesis (Fig. 4G,I). As survivin colocalises and interacts with myosin II during cytokinesis [61], we immunostained for myosin II (anti‐NMII), and this revealed that survivin and myosin II colocalised in these filaments in SVN‐GFP cells that had prematurely escaped mitosis (Figs 4J and S9). The presence of myosin II here suggests that these filaments represent a misplaced actomyosin contractile ring.

**Fig. 5:**
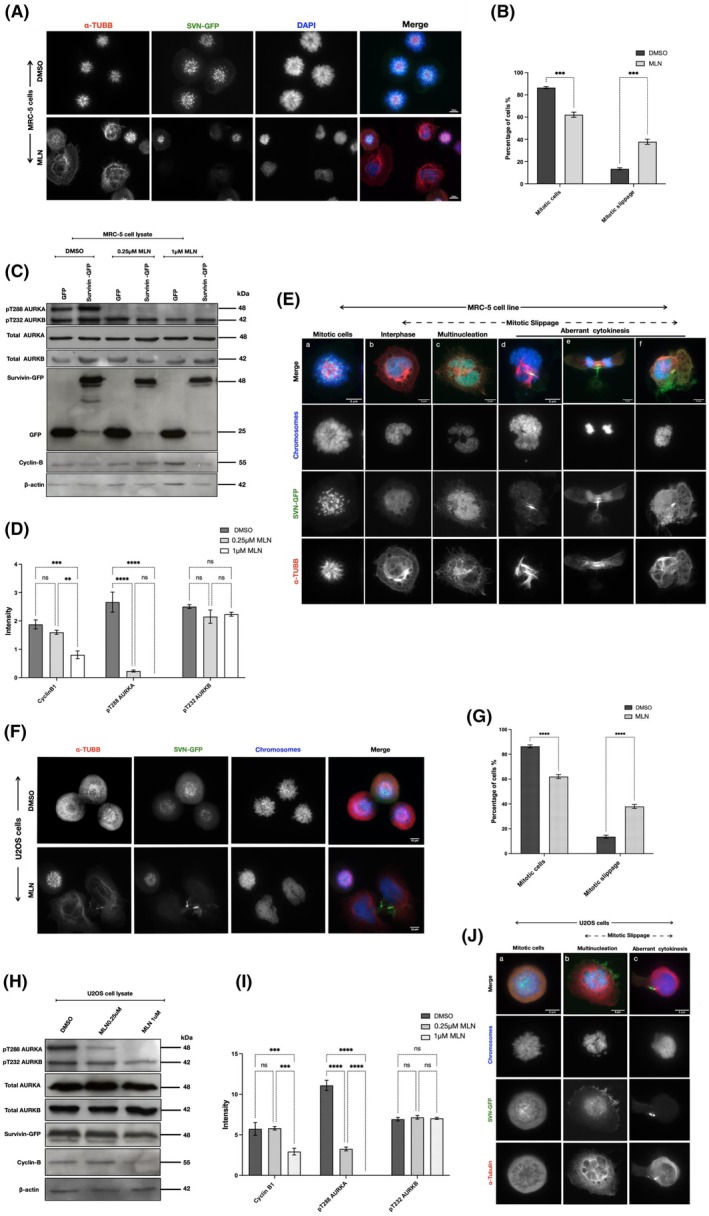
Inhibiting AURKA causes mitotic slippage in MRC5 and U2OS cells expressing SVN‐GFP. (A) Stable MRC‐5 cell line expressing survivin‐GFP was treated with DMSO (CTL), or with 1 μm MLN, as demonstrated in Fig. 4A. Cells were fixed and immunostained for α‐tubulin (α‐TUBB) (red), survivin‐GFP (SVN‐GFP) (green). DAPI stain was used to mark the chromosomes (blue). Scale bar: 10 μm. (B) Graph represents a two‐way ANOVA analysis of the quantification of mitotic and non‐mitotic cells following treatment of a stable MRC‐5 cell line expressing survivin‐GFP with DMSO or 1 μm MLN, presented in percentage. Number of mitotic cells in DMSO‐treated cells (*n*1 = 413) (*n*2 = 363) (*n*3 = 393) and in MLN‐treated cells (*n*1 = 185) (*n*2 = 167) (*n*3 = 170). Number of non‐mitotic cells in DMSO (*n*1 = 60) (*n*2 = 56) (*n*3 = 53), MLN (*n*1 = 105) (*n*2 = 120) (*n*3 = 114). (C) Immunoblot of mitotically arrested stable MRC‐5 cell lysate expressing GFP or survivin‐GFP and treated with either DMSO, 0.25 μm MLN or 1 μm MLN, as demonstrated in Fig. 4A. Immunoblotting for pThr288‐AURKA and pThr232‐AURKB was performed to assess AURKA and AURKB activity, respectively. Blots were also probed for total AURKA and AURKB. β‐actin was used as a loading control, and cyclin B1 as a mitotic marker. (D) Graphs demonstrate a two‐way ANOVA of the expression level intensity of pT288 AURKA, pT232 AURKB and cyclin B1 normalised to β‐actin in DMSO‐ and MLN‐treated MRC‐5 cells expressing survivin‐GFP. (E) Images represent a collection of MRC‐5 cells undergoing different fates following 1 μm MLN treatment. Cells were immunostained for α‐tubulin (red). DAPI stain was used to mark the nucleus/chromosomes (blue) and survivin tagged to GFP (SVN‐GFP) in (green). Scale bar: 8 μm. (F) Stable U2OS cell line expressing survivin‐GFP was treated with DMSO (CTL), or with 1 μm MLN, as demonstrated in Fig. 4A. Cells were immunostained for α‐tubulin (Red), chromosomes (blue) and Survivin‐GFP (green). Scale bar: 10 μm. (G) Graphs demonstrate two‐way ANOVA analysis of the number of mitotic and non‐mitotic U2OS cells stably expressing survivin‐GFP and treated with DMSO (CTL) or 1 μm MLN, presented in percentage. Number of mitotic cells in DMSO‐treated cells (*n*1 = 389) (*n*2 = 383) (*n*3 = 377) and in 1 μm MLN‐treated cells (*n*1 = 240) (*n*2 = 254) (*n*3 = 223). Number of non‐mitotic cells in DMSO (*n*1 = 47) (*n*2 = 42) (*n*3 = 50) and in 1 μm MLN‐treated cells (*n*1 = 140) (*n*2 = 154) (*n*3 = 152). (H) Immunoblot of mitotically arrested U2OS cell lysate expressing survivin‐GFP and treated with either DMSO, 0.25 μm MLN or 1 μm MLN. Immunoblotting for pThr288‐AURKA and pThr232‐AURKB was performed to assess AURKA and AURKB activity, respectively. β‐actin was used as a loading control, and cyclin B1 as a mitotic marker. (I) Graphs demonstrate a two‐way ANOVA of pT288 AURKA, pT232 AURKB and cyclin B1 expression intensity normalised to β‐actin in DMSO‐ and MLN‐treated U2OS cells stably expressing survivin‐GFP. (J) Images represent a collection of U2OS cells undergoing different fates following 1 μm MLN treatment. Cells were fixed and immunostained for α‐tubulin (red). DAPI stain was used to mark the genetic material (nucleus) (blue), and survivin was tagged to GFP (green). Scale bar: 8 μm. All results in panels (A–E) were obtained from MRC‐5 cells stably expressing either GFP or survivin‐GFP, and panels (F–J) from U2OS cells stably expressing survivin‐GFP, following the experimental design shown in Fig. 4A. All blots and microscopy images shown are representative of three independent repeats. Quantitative data are presented as means ± SD (*n* = 3). *P* value (ns = non‐significant, **P < 0.01, ***P < 0.001, ****P < 0.0001).

To investigate whether high levels of survivin affect mitotic cell fate in response to MLN generally and are not HeLa cell specific, we repeated the MLN experiments in MRC5 (Fig. 5A–E) and U2OS (Fig. 5F–I). The only difference was that 1 μm MLN was needed to inhibit AURKA in these lines, but importantly, this had no impact on AURKB (Fig. 5D,G). Similarly to HeLa cells, cyclin B1 expression levels dropped significantly in survivin‐GFP‐expressing MRC5 (Fig. 5C,D) and U2OS cells (Fig. 5H,I) compared to GFP‐expressing or DMSO‐treated controls, and mitotic slippage occurred (Fig. 5A,C,D,F,H,J). Collectively, these results indicate that upregulating survivin can cause premature exit and aberrant cytokinesis in both normal and transformed cells lacking active AURKA.

### 3.5 Inhibition of AURKB yields a different mitotic fate than inhibiting AURKA

**Fig. 6:**
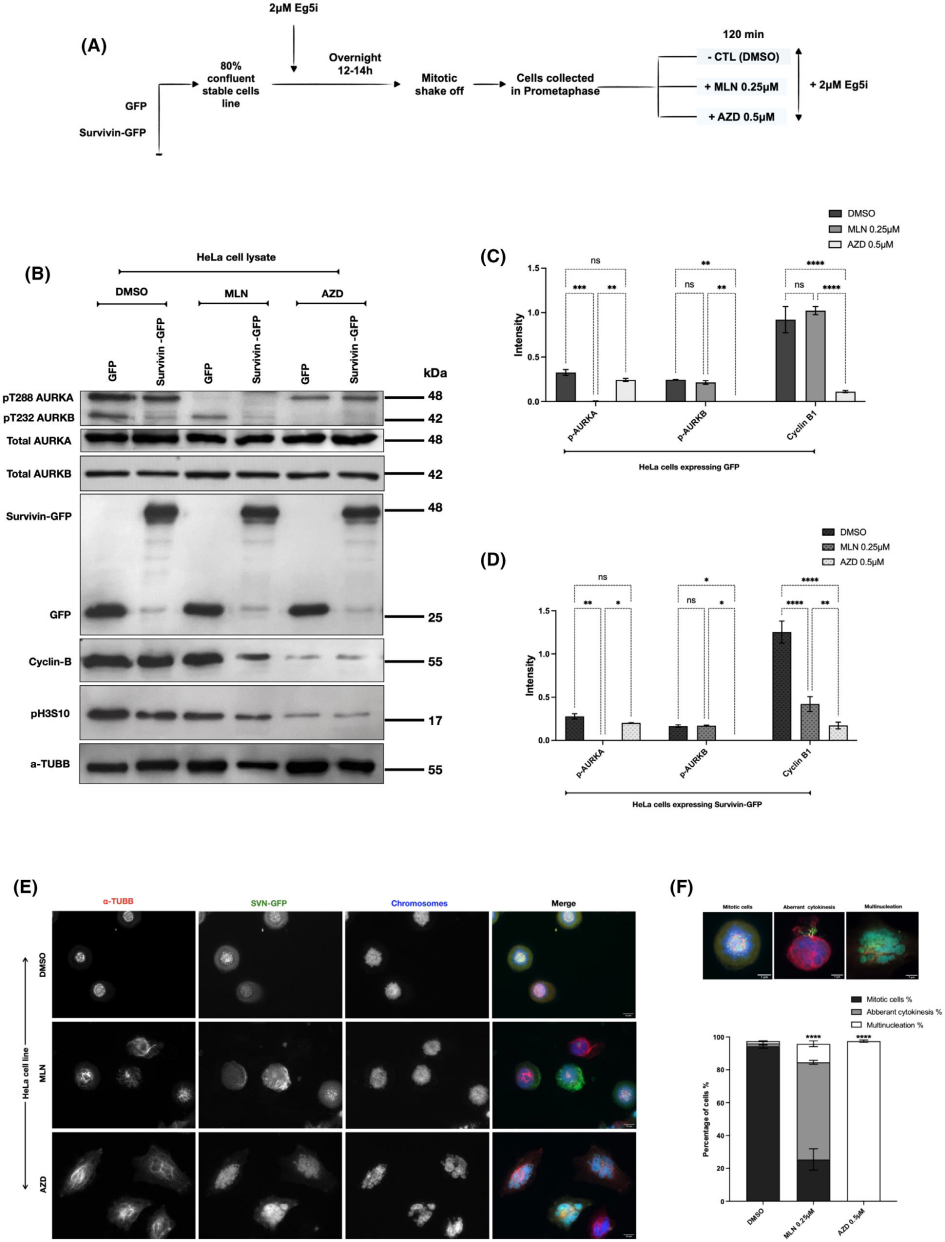
AURKB inhibition causes multinucleation. (A) Schematic demonstrating the method used for cell synchronisation and treatment. Stable HeLa cell lines expressing either GFP or survivin‐GFP were synchronised using 2 μm Eg5 inhibitor overnight. The mitotically arrested cells were treated with either DMSO, 0.25 μm MLN or 0.5 μm AZD for 120 min. (B) Immunoblot of mitotically arrested GFP or survivin‐GFP stable HeLa cell lysate treated with either DMSO (CTL), MLN or AZD. Immunoblotting against pThr288 AURKA and pThr232 AURKB was used to determine AURKA and AURKB activity, respectively. α‐tubulin (α‐TUBB) was used as a loading control and cyclin B1 as a mitotic marker. The levels of pH3S10, a substrate of AURKB, were also detected. (C, D) Graphs demonstrate a two‐way ANOVA analysis of the intensity of pT288 AURKA, pT232 AURKB and cyclin B1 normalised to α‐tubulin in DMSO (CTL), MLN and AZD‐treated stable HeLa cells expressing survivin‐GFP. (E) Images represent HeLa cells expressing survivin‐GFP and treated with DMSO, MLN and AZD. The cells were fixed and immunostained for α‐tubulin (red), DAPI stain to mark the nucleus/chromosomes (blue) and survivin tagged to GFP in (green). Scale bar: 10 μm. (F) Graph demonstrates a two‐way ANOVA analysis of the number of stable HeLa cells expressing survivin‐GFP arrested in mitosis or undergoing aberrant cytokinesis and multinucleation following treatment with DMSO (CTL), 0.25 μm MLN or 0.5 μm AZD, presented in percentage. Number of mitotic cells in cells treated with DMSO (*n*1 = 340) (*n*2 = 363), 0.25 μm MLN (*n*1 = 105) (*n*2 = 65), and 0.5 μm AZD (*n*1 = 0) (*n*2 = 0). Number of cells undergoing aberrant cytokinesis in DMSO (*n*1 = 8) (*n*2 = 5), 0.25 μm MLN (*n*1 = 210) (*n*2 = 188), and 0.5 μm AZD (*n*1 = 0) (*n*2 = 0). Number of multinucleated cells in DMSO (*n*1 = 2) (*n*2 = 3), 0.25 μm MLN (*n*1 = 35) (*n*2 = 40), 0.5 μm AZD (*n*1 = 334) (*n*2 = 350). All results in this figure were obtained from HeLa cells stably expressing either GFP or survivin‐GFP following the experimental design shown in (A). All blots and microscopy images shown are representative of three independent repeats. Quantitative data are presented as means ± SD (*n* = 3). *P* value (ns = non‐significant, *P* < 0.05, **P < 0.01, ***P < 0.001, ****P < 0.0001).

Next, as AURKB expression and activity appeared to be upregulated as a consequence of depleting AURKA (Figs 3G and 4D), we investigated whether inhibition of the two AURKs caused a similar cell fate. HeLa cells overexpressing SVN‐GFP and GFP were treated with 0.5 μm AZD, a selective inhibitor of AURKB, and its action compared to treatment with 0.25 μm MLN was examined (see Fig. 6A). Immunoblotting with antibodies to detect pT228 AURKA and pT232 AURKB, as well as total AURKA/B, proved the specificity of these treatments (Fig. 6B–D). Interestingly, anti‐cyclin B1 level was reduced in both GFP‐ and SVN‐GFP‐expressing cells upon AZD treatment, whereas MLN only caused reduction in cyclin B1 in cells with SVN‐GFP cells (Fig. 6B–D). Moreover, when examined using fluorescence imaging, the phenotypic characteristics were very different: approximately 60% of the MLN‐treated SVN‐GFP cells underwent aberrant cytokinesis, forming a single large aggregation of DNA, whereas 97% of the cells treated with AZD formed multiple DNA entities/micronuclei (Fig. 6E,F). Thus, although both AURKA and AURKB cause mitotic slippage, these data suggest that the underlying mechanisms involved are different.

### 3.6 AURKA activity is required to maintain centromeric survivin and for BubR1 recruitment to the kinetochores in prometaphase

**Fig. 7:**
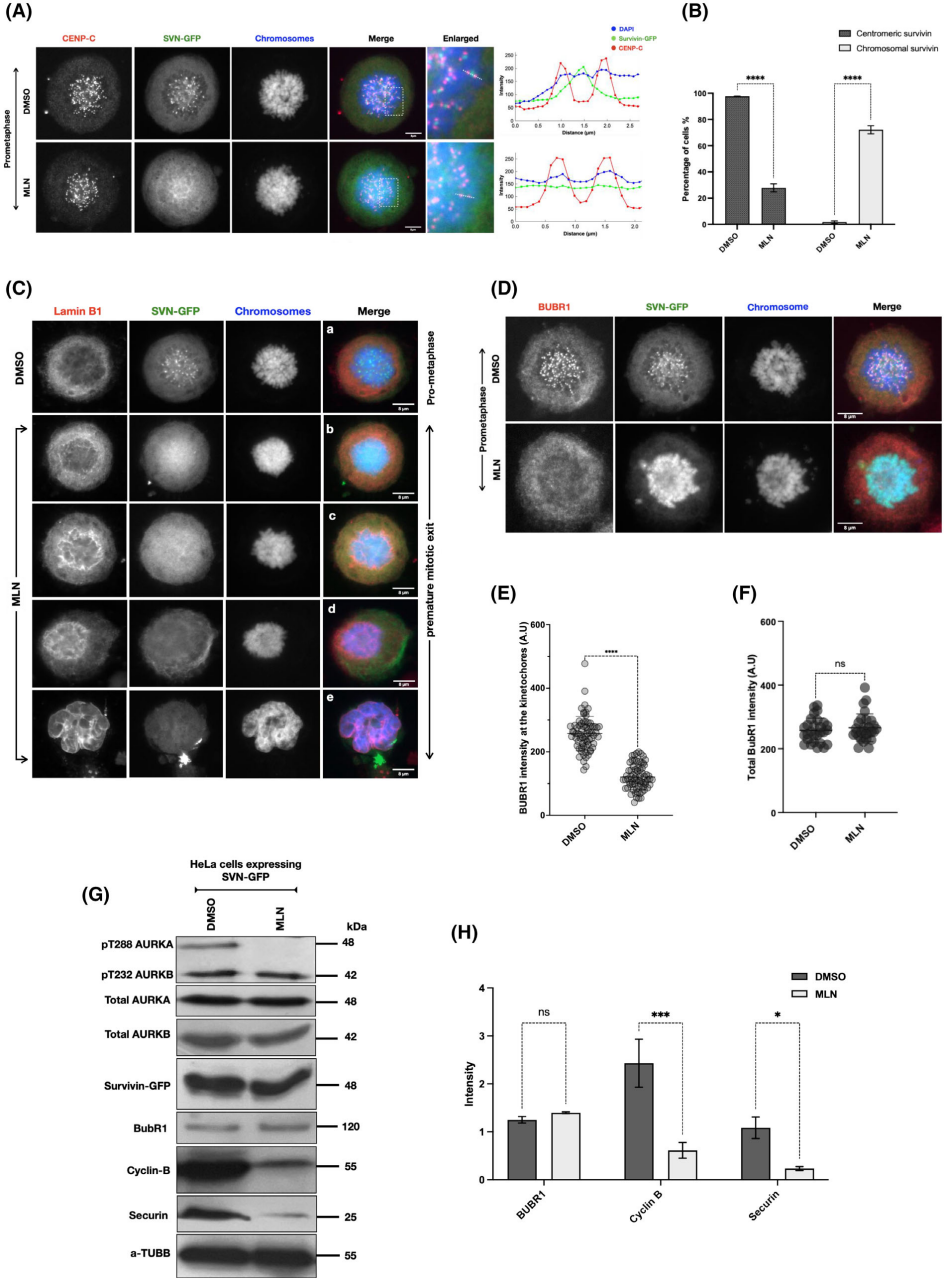
AURKB inhibition enables cells with high levels of survivin to breach the SAC. (A) Stable HeLa cell line expressing survivin‐GFP and treated with DMSO (CTL) or 0.25 μm MLN as illustrated in Fig. 4A. Cells were fixed and immunostained for CENP‐C (red), DAPI stain for chromosomes (blue), and survivin‐GFP in (green). Scale bar: 8 μm. Fluorescence intensity profiles were obtained along the white dashed lines in the selected centromere region of cells under different conditions, DMSO and MLN. Scale bars = 8 μm. (B) Graph demonstrates two‐way ANOVA analysis of the percentage of cells with normal localisation of survivin (centromeric survivin) and cells with mislocalisation of survivin (chromosomal survivin) during prometaphase arrest following treatment with DMSO or MLN. Number of cells with centromeric survivin in DMSO‐treated cells (*n*1 = 213) (*n* = 154) and in MLN‐treated cells (*n*1 = 75) (*n* = 57). Number of cells with chromosomal survivin in DMSO‐treated cells (*n*1 = 2) (*n* = 1) and in MLN‐treated cells (*n*1 = 200) (*n* = 164). (C) Images represent a collection of stable HeLa cells expressing survivin‐GFP and undergoing mitotic slippage following 0.25 μm MLN treatment. Cells were immunostained for lamin B (red). DAPI stain was used to mark the nucleus/chromosomes (blue) and survivin‐GFP (green). Panel (a) represents a prometaphase‐arrested cell treated with DMSO (CTL). Panels (b–e) show cells treated with MLN and going through premature mitotic exit. Scale bar: 8 μm. (D) Prometaphase‐arrested stable HeLa cells expressing survivin‐GFP and treated with DMSO or 0.25 μm MLN were immunostained against BubR1 as presented in (red), chromosomes in (Blue), survivin‐GFP in (green). Scale bar: 8 μm. (E, F) Graphs demonstrated Student's *t‐*test analysis of the differences in the mean intensity of total BubR1 presented in (F) or at the unattached kinetochores presented in (E) of mitotically arrested cells treated with DMSO or with 0.25 μm MLN. (G) Immunoblot of mitotically arrested stable HeLa cells expressing survivin‐GFP treated with either DMSO or 0.25 μm MLN. Cells were immunoblotted against BubR1, securin and cyclin B1. pThr288 AURKA and pThr232 AURKB were used to determine AURKA and AURKB activity, respectively. α‐tubulin was used as a loading control. (H) Graph demonstrates two‐way ANOVA analysis of the mean expression intensity of BubR1, cyclin B1 and securin, normalised to α‐tubulin, in stable HeLa cells expressing survivin‐GFP treated with either DMSO or 0.25 μm. All results in this figure were obtained from HeLa cells stably expressing survivin‐GFP following the experimental design shown in Fig. 4A. All blots and microscopy images shown are representative of three independent repeats. Quantitative data are presented as means ± SD (*n* = 3). *P* value (ns = non‐significant, *P < 0.05, ***P < 0.001, ****P < 0.0001).

Similarly to the effect of AURKA depletion on endogenous survivin localisation (Fig. 3A), rather than focusing on the CENP‐C highlighted centromeres (red), when AURKA was inhibited, SVN‐GFP was located along the chromosome arms (Fig. 7A). Indeed, 73% of the AURKA‐inhibited prometaphase cells had survivin spread over the chromosome arms compared to 3% when AURKA was active (Fig. 7B).

Having witnessed mitotic slippage of SVN‐GFP cells when AURKA was inhibited, we next asked whether the localisation of survivin is correlated with the observed characteristics of G1. For this, SVN‐GFP cells were immunostained for lamin B to view any nuclear envelope formation [62] and cyclin B1 to evidence CDK1 inactivation [63]. As shown in Fig. 7C, Lamin B was diffusely localised in the cytoplasm of cells treated with MLN when survivin was centromeric; however, it accumulated around the DNA (blue) when survivin was spread along the chromosome arms.

Since survivin regulates AURKB function by mediating its localisation at the centromeres [19, 20, 21, 22], and this is vital for SAC recruitment during early mitosis [64], the integrity of BubR1 in cells lacking active AURKA was investigated. As demonstrated in Fig. 7D,E and Fig. S10, immunostaining of SVN‐GFP HeLa cells treated with MLN revealed that BubR1 was significantly reduced at the kinetochores when survivin was mis‐localised, albeit the overall expression of BubR1 remained unchanged (Fig. 7F–H). These data suggest that the mitotic slippage observed was due to a decrease in BubR1 affinity towards the unattached kinetochores, rather than a drop in its expression levels.

Having witnessed premature cyclin B degradation, BubR1 mislocalisation, and signs of G1 entry, we asked whether securin, the APC/C target that needs to be degraded to allow chromosome segregation to occur, had also been degraded. Using the same experimental schema shown in Fig. 4A, similarly to cyclin B1, securin expression level was found to be significantly reduced in MLN‐treated cells but still abundantly present in control cells (Fig. 7G,H, Fig. S11A–D).

Collectively, these data suggest that premature removal of AURKA inactivation causes cells with high levels of survivin to breach the BubR1‐mediated SAC within 2 h, resulting in premature APC/C activation and consequently degradation of cyclin B and securin, ultimately yielding a single viable, polyploid cell through mitotic slippage.

## 4 Discussion

Survivin is an adaptor protein that brings other proteins and enzymes into contact to perform their duties in multiple signalling pathways [65, 66]. As part of the CPC, it plays an essential role in mitosis, and as an inhibitor of apoptosis protein (IAP), it can inhibit cell death. Pathologically, it is upregulated in all cancers [67], and thus it is an attractive target for oncologists.

AURKA is a mitotic serine/threonine kinase with oncogenic properties. It is considered a promising target for cancer therapy and a predictive marker for cancer [68]. Elevation in its expression correlates with cancer progression and resistance to chemotherapy [69, 70, 71]. In mammals, AURKA belongs to the aurora kinase family (AURKs), which has three members, A, B, and C. To date, only AURKB and AURKC have been shown to interact with survivin, which they do via CPC integration during mitosis and meiosis, respectively [72, 73, 74]. However, the discovery of a pool of AURKA at the centromeres during prometaphase [12, 14, 48] and its ability to phosphorylate similar centromeric substrates to AURKB [51, 52] raised the question of potential redundancy between AURKA and B in mitosis. In line with this, here we noted that AURKB expression and activity increased when AURKA levels were depleted, indicating a compensatory mechanism may exist between them, as has been suggested previously [5, 75].

However, in our view, the hypothesis that AURKA and B may be functionally redundant was overturned by three main findings. First, AURKA, similarly to AURKB, regulates the phosphorylation levels of H3T3 and consequently survivin recruitment [14, 22, 76, 77]. However, although AURKB levels and activity increased when AURKA was depleted (Fig. 3G,H), survivin mislocalised in its absence (Fig. 3A). This indicates that the presence of AURKA is required for proper localisation of survivin during early mitosis. Furthermore, although the relocation of the CPC to the central spindles during anaphase requires the formation of a complex between the kinesin‐6 family motor protein (MKlp2), AURKB, and INCENP [78], we observed that the transfer of survivin to the central midzone at anaphase was also disrupted when AURKA was depleted, even though AURKB was still active (Fig. 3E). Although AURKA is known to regulate Mklp1 recruitment to the central spindles [79], to our knowledge, this report is the first to show that survivin transfer to the central anaphase spindle is also AURKA dependent.

Second, this study revealed that the AURKA interaction sites within survivin are distinct from those of AURKB: AURKB interacts via D^70^D^71^ residues in the BIR domain [55], while the AURKA interaction site is located somewhere between amino acids 30 and 60, which offers the possibility of simultaneous, rather than competitive or compensatory, binding between survivin and AURKA and B (Fig. 2I, Fig. S2).

However, while our data do not directly address whether AURKA forms a complex similar to the established CPC, previous studies have shown that AURKA can bind to INCENP [48, 80], a core CPC component that also interacts with the C‐terminus of survivin [17]. Given that AURKA, INCENP and survivin all localise to the centromeres during early mitosis [13, 14, 19, 20, 21], and that they each interact with the C terminus of survivin, we propose that INCENP could act as a bridging factor mediating the interaction between AURKA and survivin. Whether borealin also plays a role in this context remains unclear and would require additional investigation focused on complex formation.

Moreover, although AURKA and survivin are both present in interphase, their localisation differs. AURKA is mostly centrosomal, where it contributes to centrosome maturation and spindle formation [81]. However, a smaller pool of AURKA can also be detected in the cytoplasm and nucleus [82]. Survivin, on the other hand, is found in both the nucleus and cytoplasm, and its localisation is regulated by CRM1‐dependent nuclear export [83, 84]. Thus, although they can interact directly, they may not be in close enough proximity to do so in interphase.

Third, although inhibition of both AURKs caused mitotic slippage, the phenotypic outcomes and their sensitivities to the levels of survivin expression were different. AURKA inhibition caused mitotic slippage only in cells with high levels of survivin, and this resulted in an undivided cell with a single polyploid nucleus and mislocalised cytokinetic ring proteins. We speculate that this aneuploid cell could remain viable for some time before eventually succumbing to apoptosis via the ‘tetraploidy checkpoint’ [85, 86]. However, despite observing few arrested HeLa cells undergoing apoptosis when AURKA was inhibited, the percentage of cells with this fate was consistent with the DMSO control, indicating that this was independent of AURKA activity. One possible explanation is that premature mitotic exit occurs via gradual ubiquitination of cyclin B1 by APC despite the presence of an active mitotic checkpoint [87]. Thus, upregulating the levels of survivin induces proliferation [88, 89] and inhibits apoptosis via antagonising the enzymatic activity of caspases [90, 91, 92], leading to rapid mitotic slippage before the apoptotic signals reach a threshold required for inducing cell death.

In this study, AURKA activity was also found to be required for proper centromeric localisation of survivin during prometaphase, and a complete mislocalisation of survivin at the chromosomes was correlated with a reduction in BubR1 levels at the kinetochores, cyclin B1 inhibition, and the reassembly of the lamina, which is an indicator of the inhibition of the phosphorylation of lamins such as lamin B due to a drop in cyclin B1 levels and CDK1 inactivation [63]. This suggests that although survivin localisation is regulated by both AURKA/B [14, 77], AURKB alone is insufficient to achieve full CPC recruitment to the centromere.

In contrast to AURKA inhibition, inactivation of AURKB resulted in one large cell with multiple micronuclei, a morphological feature of mitotic catastrophe [93], which is consistent with Tsuda et al. [94] and McHugh et al. [95]. Moreover, we found that this outcome was the same regardless of survivin status. During prometaphase, AURKB plays a vital role in correcting malorientated chromosomes by negatively regulating the MT catastrophe factor, mitotic centromere‐associated kinesin (MCAK) [96, 97, 98, 99]. In this role, AURKB stabilises correctly connected kinetochore MTs by phosphorylating MCAK at the centromere. From the different phenotypic outcomes, our data suggest that AURKA does not aid in chromosome error correction.

**Fig. 8:**
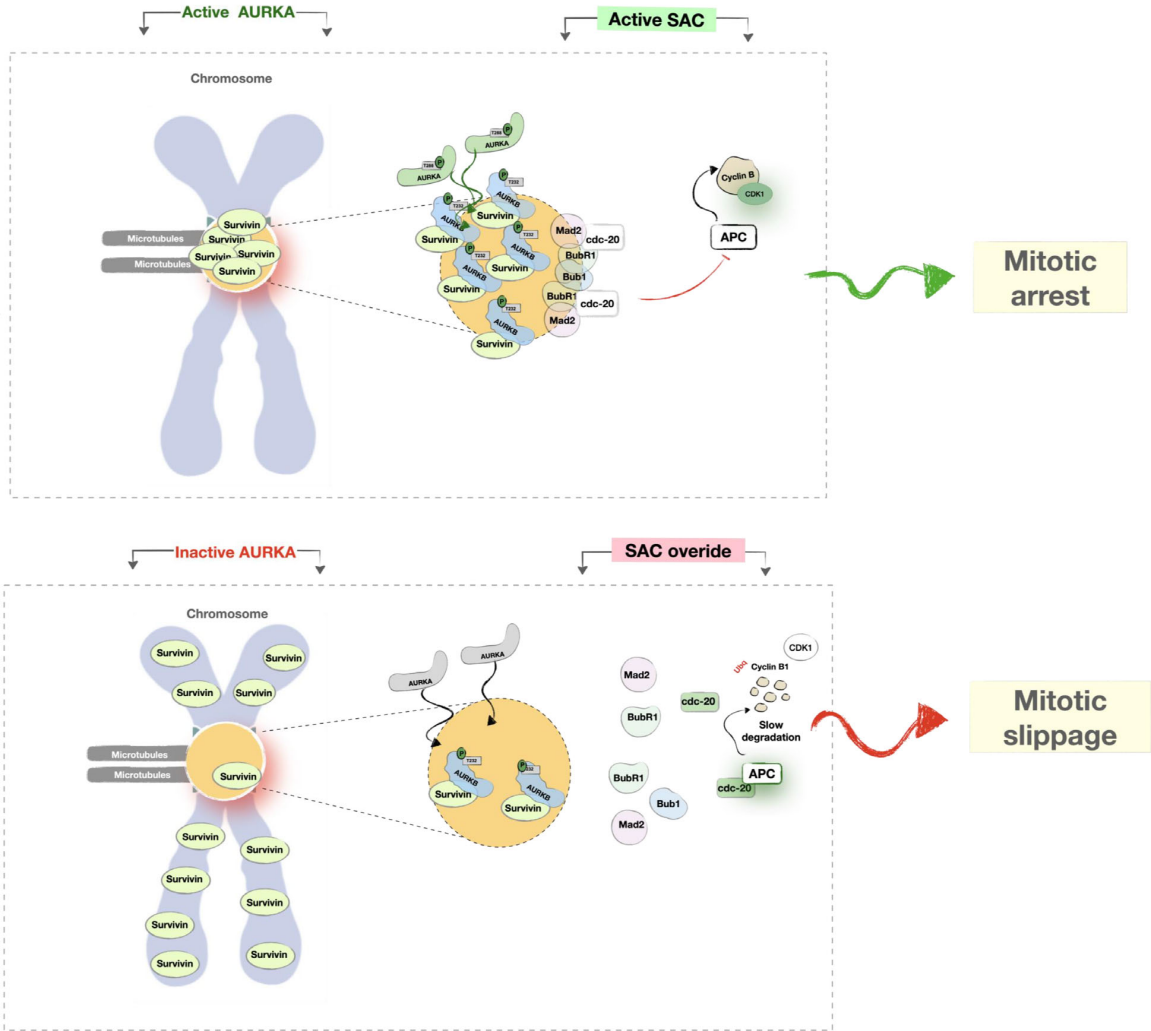
Overview of the hypothesised mechanism behind mitotic slippage caused by AURKA inactivation. AURKA regulates SAC function via regulating survivin centromeric localisation. The presence of sufficient phosphorylation levels of AURKA maintains survivin localisation at the centromeres and consequently AURKB centromeric localisation and its function as a regulator of SAC. AURKA inactivation causes premature removal of survivin from the centromeres, leading to a reduction in the levels of AURKB accumulated at the centromeric region, which eventually causes cells to escape mitotic arrest due to a reduction in the levels of SAC accumulated at the centromeres.

We also found that inhibiting AURKA, similarly to AURKB [42, 64], causes premature mitotic exit in cells overexpressing survivin, via disrupting the levels of BubR1 localised at the kinetochores. However, since survivin regulates AURKB function and localisation at the centromeres [19, 20, 21, 22, 100], the current study suggests that AURKA activity regulates cell fate via maintaining survivin localisation and function as a regulator of AURKB during prometaphase. These findings are supported by Yu et al. [14], which suggested that AURKA activity is required for SAC recruitment at the G2‐M transition phase via regulating AURKB recruitment at the kinetochores. Collectively, we conclude that AURKA is crucial to maintain the SAC by ensuring correct survivin and CPC localisation at the centromeres in early mitosis, and that without this focus, BubR1 recruitment to centromeres is impaired, enabling premature APC/C activation and mitotic slippage, which yields a single viable cell with one polyploid nucleus, as illustrated in Fig. 8.

In summary, we have shown for the first time that AURKA interacts with survivin and regulates its function during both early and late mitosis and that deregulating survivin expression and AURKA activity redirects cell fate in favour of mitotic slippage, leading to genetic instability. These results suggest that the efficacy of AURKA‐targeted anticancer drugs like MLN8237 (Alisertib) may be compromised in tumours with elevated survivin expression. In this regard, newer AURKA inhibitors such as LY3295668, with enhanced selectivity and potency, could offer improved outcomes in certain tumour contexts [101].

## Conflict of interest

The authors declare no conflict of interest.

## Author contributions

SPW and HA conceived and designed the project. HA acquired the data with assistance from SWA, particularly in the acquisition of independent repeats and at revision. HA and SPW wrote the paper.

## Supporting information


**Fig. S1.** The synchronisation method used to collect cells in different stages of mitosis.
**Fig. S2.** Survivin interaction site with AURKA is located within the 30–60 amino acids of the BIR domain.
**Fig. S3.** Optimisation of the AURKA knockdown using siRNA against AURKA.
**Fig. S4.** Knocking down AURKA causes survivin mislocalisation during prometaphase.
**Fig. S5.** Knocking down AURKA causes spindle defects and survivin mislocalisation during mitosis.
**Fig. S6.** Knocking down AURKA causes mislocalisation of AURKB during early mitosis. A gallery of cells treated with either 40 nm scrambled‐siRNA (siControl) or 40 nm AURKA‐siRNA (siAURKA) for 48 h. Treated cells were fixed and immunostained for AURKB to examine its localisation. Panels (a, c, e and g) immunostained for AURKA (green), survivin (red) and chromosomes (blue) and panels (b, d, f and h) immunostained for AURKB (green), survivin (red) and chromosomes (blue). Scale bar: 7 μm. All the microscopy images shown are representative of three independent repeats.
**Fig. S7.** Knocking down AURKA causes mislocalisation of CPC members INCENP and borealin during early mitosis.
**Fig. S8.** Inhibiting AURKA phosphorylation using different concentrations of MLN and assessing survivin overexpression.
**Fig. S9.** Inhibiting AURKA activity caused cells to exit mitosis prematurely via forming an aberrant contractile ring.
**Fig. S10.** AURKA inhibition causes a decrease in the levels of BubR1 at the unattached kinetochores.
**Fig. S11.** AURKB inhibition enables cells with high levels of survivin to breach the SAC in MRC5 and U2OS cells.

**Table S1.** Plasmid list.

## Data Availability

All supporting data are available in the Supporting Information. Reagents generated for this paper are available from the corresponding author (SPW) upon request.
