# Supplementary material for: Survivin and Aurora Kinase A control cell fate decisions during mitosis
Source: Mol Oncol. 2025 Oct 12;20(3):727–52. doi: 10.1002/1878-0261.70141 (PMC13042919; doi:10.1002/1878-0261.70141)
Supplement: Supplementary file 1 — Fig. S1. The synchronisation method used to collect cells in different stages of mitosis. Fig. S2. Survivin interaction site with AURKA is located within the 30–60 amino acids of the BIR domain. Fig. S3. Optimisation of the AURKA knockdown using siRNA against AURKA. Fig. S4. Knocking down AURKA causes survivin mislocalisation during prometaphase. Fig. S5. Knocking down AURKA causes spindle defects and survivin mislocalisation during mitosis. Fig. S6. Knocking down AURKA causes mislocalisation of AURKB during early mitosis. A gallery of cells treated with either 40 nm scrambled‐siRNA (siControl) or 40 nm AURKA‐siRNA (siAURKA) for 48 h. Treated cells were fixed and immunostained for AURKB to examine its localisation. Panels (a, c, e and g) immunostained for AURKA (green), survivin (red) and chromosomes (blue) and panels (b, d, f and h) immunostained for AURKB (green), survivin (red) and chromosomes (blue). Scale bar: 7 μm. All the microscopy images shown are representative of three independent repeats. Fig. S7. Knocking down AURKA causes mislocalisation of CPC members INCENP and borealin during early mitosis. Fig. S8. Inhibiting AURKA phosphorylation using different concentrations of MLN and assessing survivin overexpression. Fig. S9. Inhibiting AURKA activity caused cells to exit mitosis prematurely via forming an aberrant contractile ring. Fig. S10. AURKA inhibition causes a decrease in the levels of BubR1 at the unattached kinetochores. Fig. S11. AURKB inhibition enables cells with high levels of survivin to breach the SAC in MRC5 and U2OS cells. [file MOL2-20-727-s001.pdf]

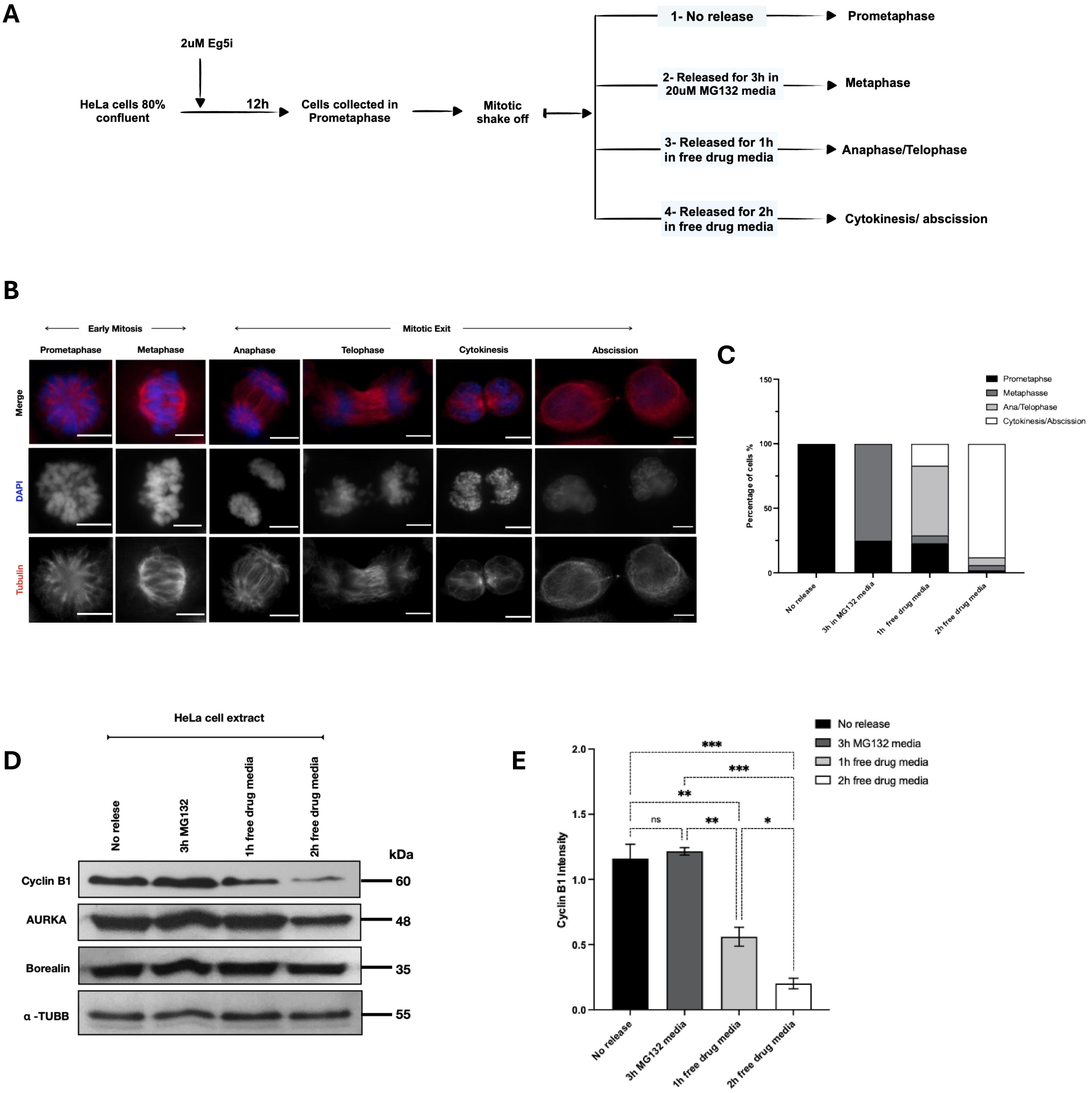

Fig. S1: The synchronisation method used to collect cells in different stages of mitosis.
(A) Diagram demonstrating the method used to synchronise cells in different stages of mitosis. HeLa cells were arrested using 2 μM Eg5 inhibitor overnight. The prometaphase-arrested cells were divided into four groups. The first group of cells was collected with no release to obtain cells in the prometaphase stage. The second group was released in 20 μM MG132 for 3 h to obtain cells in metaphase. The third and the fourth groups were released in a drug-free medium for either 1 h or 2 h to obtain cells in the anaphase/telophase stage or the cytokinesis/abscission phase, respectively. **(B)** A gallery of HeLa cells treated with 2 μM Eg5 inhibitor, seeded on poly-lysine coverslips, and released in parallel as explained in Fig. S1A. The cells were immunostained for α-tubulin (red), survivin (green) and the chromosomes in blue. Each image represents one cell of each mitotic stage population. Scale bar: 8 μm. **(C)** Graph shows the percentage distribution of HeLa cells at different stages of mitosis for each synchronisation method. The total number of cells counted in each group (n=200). No release (prometaphase, n=200; metaphase, n=0; ana/telophase, n=0; cytokinesis/abscission, n=0). 3 h release in MG132 (prometaphase, n=50; metaphase, n=150; ana/telophase, n=0; cytokinesis/abscission, n=0). 1 h release in free drug media (prometaphase, n=46; metaphase, n=12; ana/telophase, n=108; cytokinesis/abscission, n=34). 2 h release in free drug media (prometaphase, n=4; metaphase, n=8; ana/telophase, n=12; cytokinesis/abscission, n=176). **(D)** Western blot demonstrates the expression levels of AURKA and cyclin B1 in HeLa cells upon different methods of cell synchronisation. α-tubulin was used as a loading control and cyclin B1 as a mitotic marker. **(E)** Graph demonstrates ordinary one-way ANOVA analysis of cyclin B1 intensity, normalised to tubulin, in cell lysates collected from the different synchronisation methods. **(F)** Graph demonstrates ordinary one-way ANOVA analysis of AURKA intensity, normalised to tubulin. All blots and microscopy images shown are representative of three independent repeats. Quantitative data are presented as means ± SD (n =3). *P* value (ns = non-significant, **P* < 0.05, ***P* < 0.01, ****P* < 0.001).

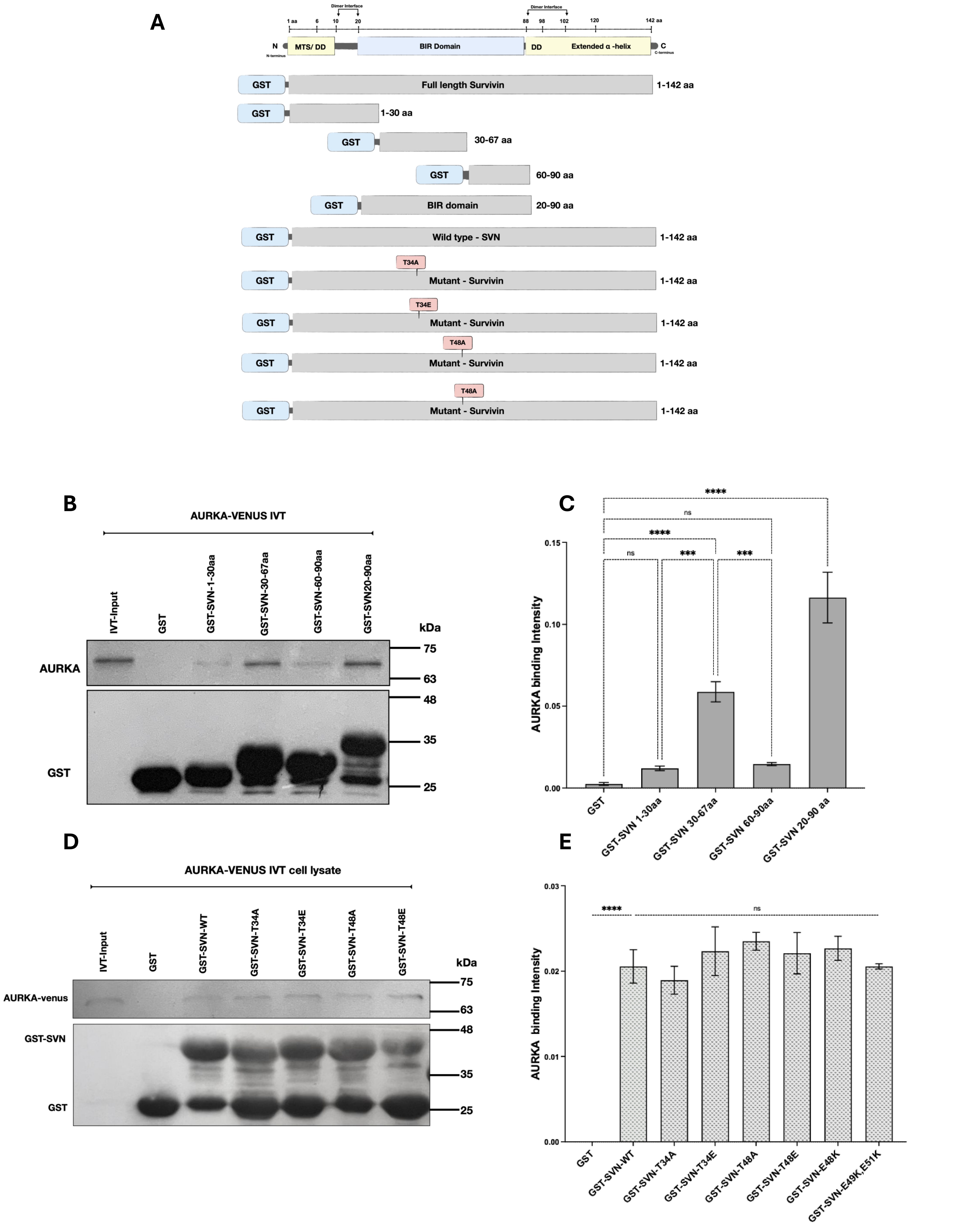

Fig. S2: Survivin interaction site with AURKA is located within the 30-60 amino acids of the BIR domain.
(A) Schematic representation of survivin structure and the GST-purified truncations used in the in vitro TNT-T7 coupled reticulocyte assay to map the direct interaction of AURKA with the BIR domain of survivin. The GST-fused proteins used in this investigation were GST-SVN 1-30 aa, GST-SVN 30- 67 aa, GST-SVN 60-90 aa, GST-SVN 20-90 aa (BIR domain), mutant survivin represented in GST-SVN T34A, GST-SVN T34E, GST-SVN T48A and GST-SVN T48E. **(B)** Immunoblot demonstrates the affinity of the *in vitro* translated AURKA towards the different truncations of the N-terminus and the BIR domain of survivin. **(C)** Graph demonstrates ordinary one-way ANOVA analysis of the intensity of the *in vitro* translated AURKA bound directly to the different truncations of the N-terminus of survivin-GST compared to the negative control, GST only. **(D)** Immunoblot demonstrates the affinity of the in vitro translated AURKA towards wild-type survivin compared to mutant survivin. **(E)** Ordinary one-way ANOVA analysis of the intensity of the in vitro translated AURKA bound directly to the different mutant versions of full-length survivin compared to wild-type survivin. AURKA intensity was normalised to GST band intensity. All blots shown are representative of three independent repeats. Quantitative data are presented as means ± SD (n =3). *P* value (ns = non-significant, ***P* < 0.001, **** *P* < 0.0001).

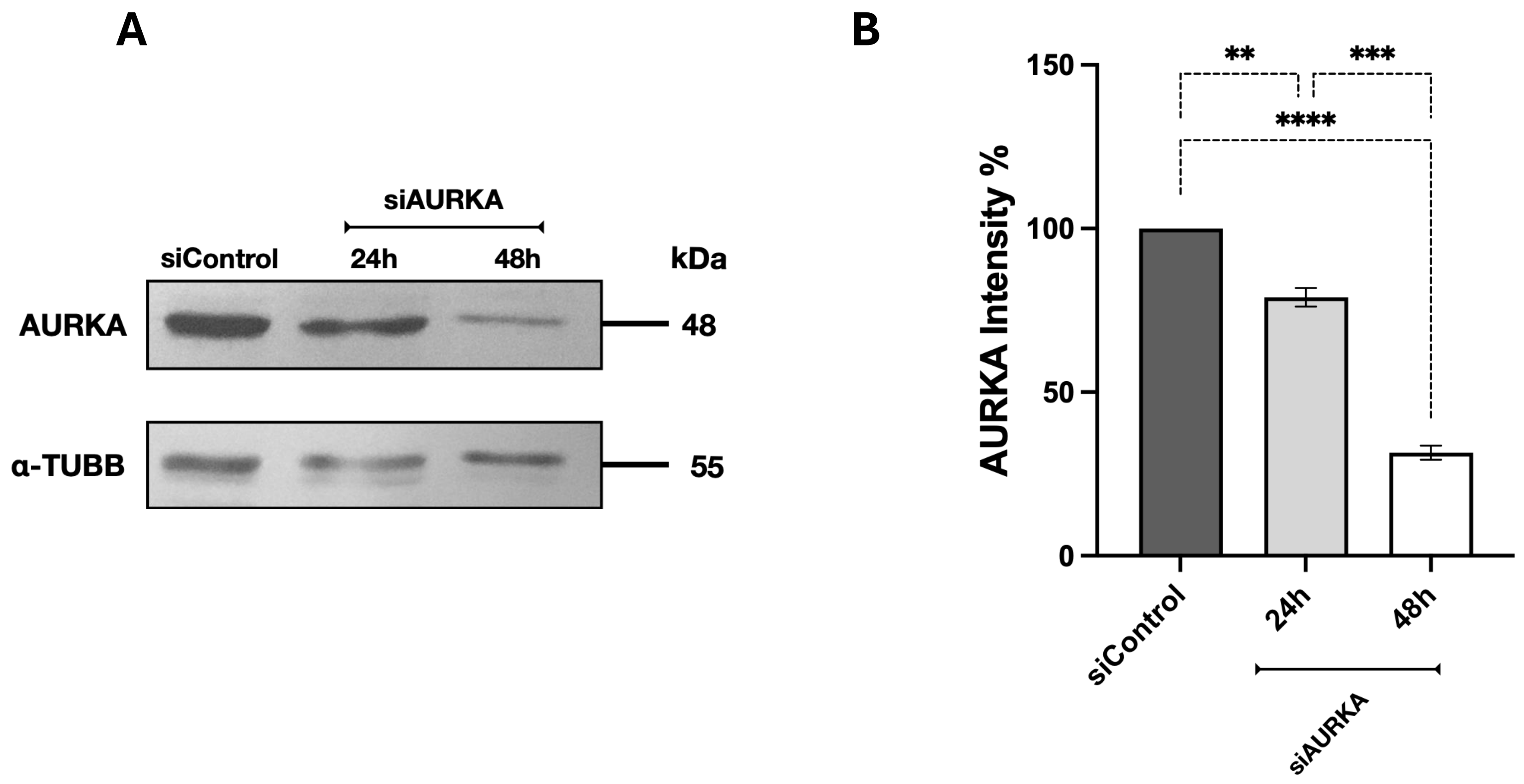

Fig. S3: Optimisation of the AURKA knockdown using siRNA against AURKA.
(A) Immunoblot demonstrates the efficiency of AURKA knockdown using HeLa cells treated with either 40 nM scrambled siRNA (negative control) or with 40 nM AURKA siRNA in two different conditions, 24 and 48 h. The treated cells were lysed, and the expression levels of AURKA were examined using western blot. **(B)** Graph demonstrates an ordinary one-way ANOVA analysis of the efficiency of AURKA knockdown at different treatment conditions as explained in S3A. The blot shown is a representative of three independent repeats. Quantitative data is presented as means ± SD (n =3). *P* value (ns = non-significant, $^{**}P < 0.01$, $^{***}P < 0.001$)

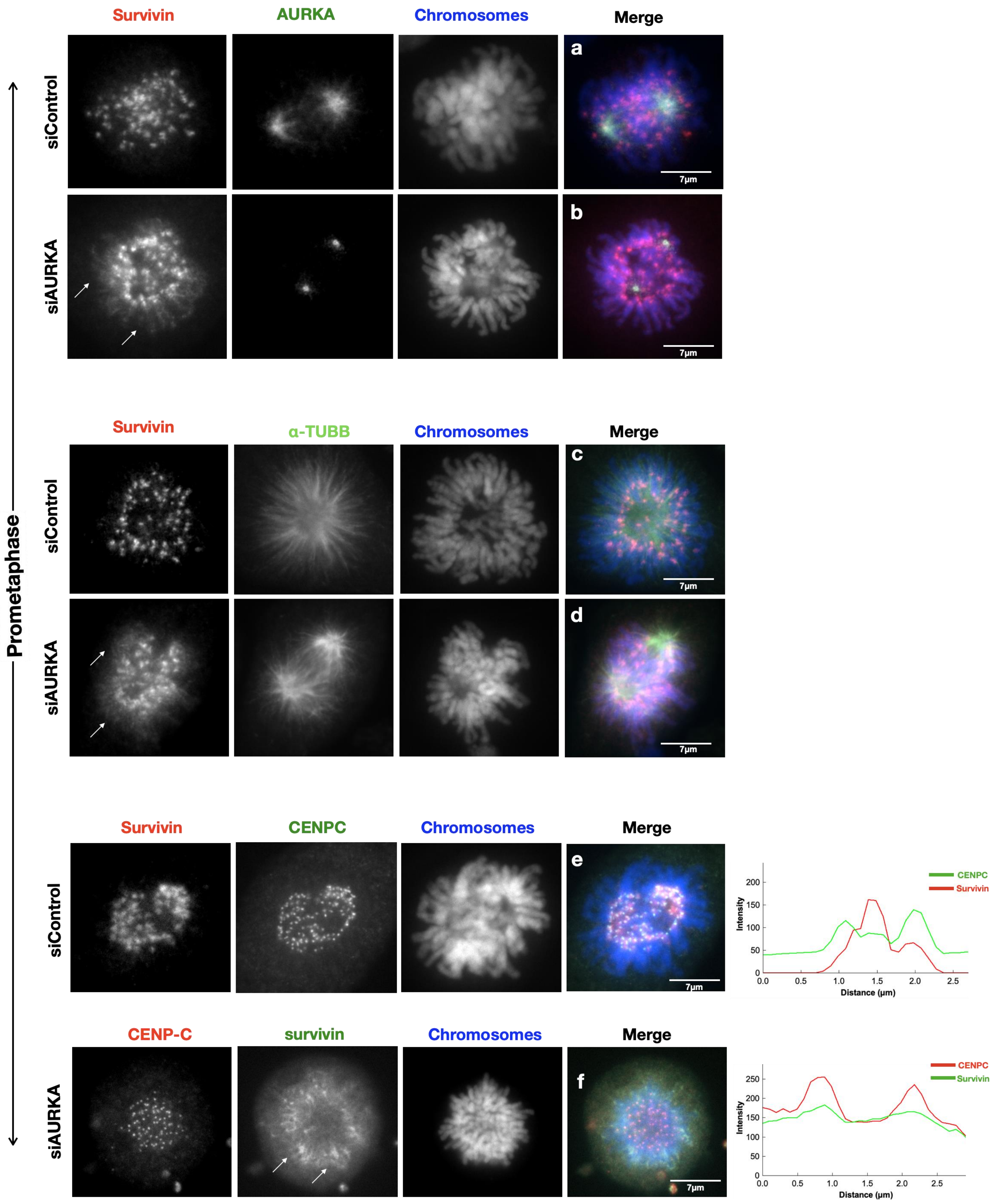

Fig. S4: Knocking down AURKA causes survivin mislocalisation during prometaphase.
Images of prometaphase HeLa cells were treated with either 40 nM scrambled siRNA (siControl) or 40 nM AURKA-siRNA for 48 hours. Cells were fixed and immunostained to examine survivin localisation. Panels (a, b) AURKA (green), survivin (red) and chromosomes (blue), (c, d) α-tubulin (green), survivin (red) and chromosomes (blue), panels (e, f) survivin (green), CENP-C (red) and chromosomes (blue). White arrows indicate survivin localisation at the chromosome arms. Intensity profiling in panels (e) and (f) was performed along centromeric regions to assess changes in survivin localisation in siAURKA-treated cells, using CENP-C as a marker. All the microscopy images shown are representative of three independent repeats.

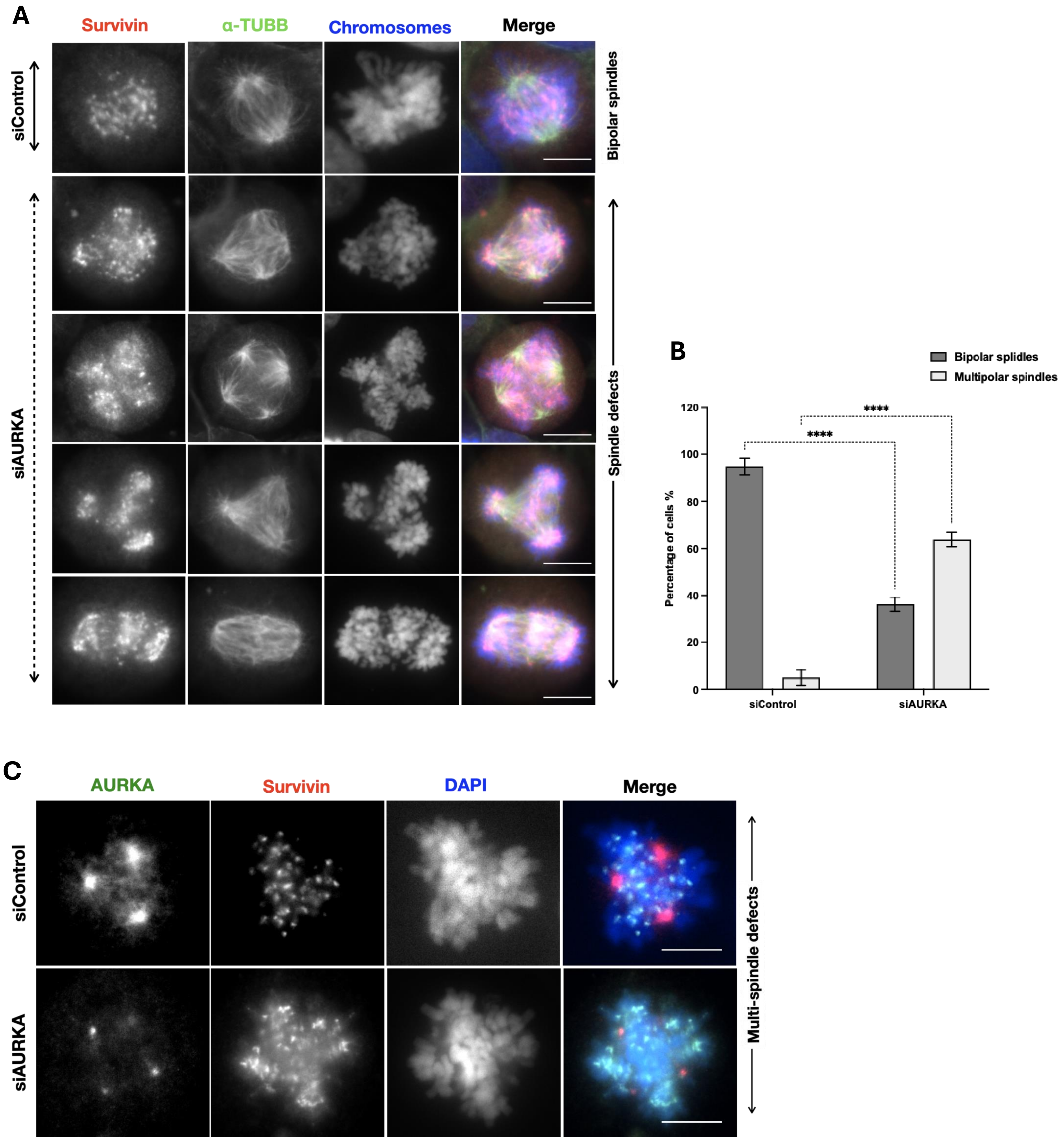

Fig. S5: Knocking down AURKA causes spindle defects and survivin mislocalisation during mitosis. (A) Images demonstrating mitotic spindle defects in HeLa cells treated with either 40 nM scrambled-siRNA (siControl) or 40 nM AURKA-siRNA for 48 h. α-tubulin (green), survivin (red) and chromosomes (blue). Scale bar: 7μm. **(B)** Graph demonstrates two-way ANOVA analysis of the number of cells experiencing bipolar spindles in siControl compared to siAURKA-treated HeLa cells. The number of cells with bipolar spindles in siControl (n1= 29) (n2= 20) (n3=34) and in siAURKA (n1= 10) (n2= 13) (n3=7). Number of cells with multipolar spindles in siControl (n1= 1) (n2= 2) (n3=1) and in siAURKA (n1= 18) (n2= 20) (n3=15). **(C)** Images demonstrate differences in survivin localisation in prometaphase cells enduring spindle defects when treated with 40 nM AURKA-siRNA compared to 40 nM scrambled-siRNA (siControl) for 48 h. AURKA (red), survivin (green) and DAPI stain for the chromosomes (blue). Scale bar: 7 μm. All microscopy images shown are representative of three independent repeats. Quantitative data are presented as means ± SD (n =3). *P* value = (**** p<0.0001).

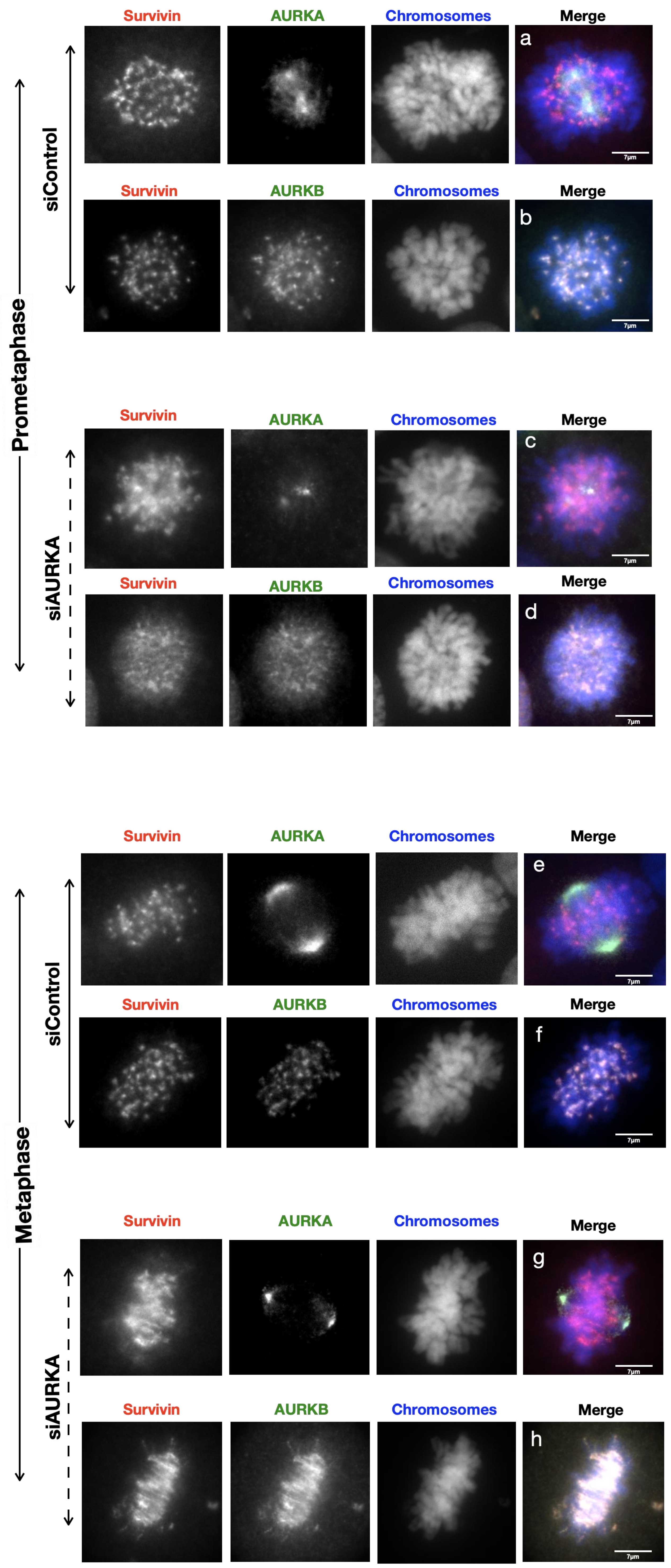

Fig. S6: Knocking down AURKA causes mislocalisation of AURKB during early mitosis.

A gallery of cells treated with either 40 nM scrambled-siRNA (siControl) or 40 nM AURKA-siRNA (siAURKA) for 48 h. Treated cells were fixed and immunostained for AURKB to examine its localisation. Panels (a, c, e and g) immunostained for AURKA (green), survivin (red) and chromosomes (blue) and panels (b, d, f and h) immunostained for AURKB (green), survivin (red) and chromosomes (blue). Scale bar: 7 µm. All the microscopy images shown are representative of three independent repeats.

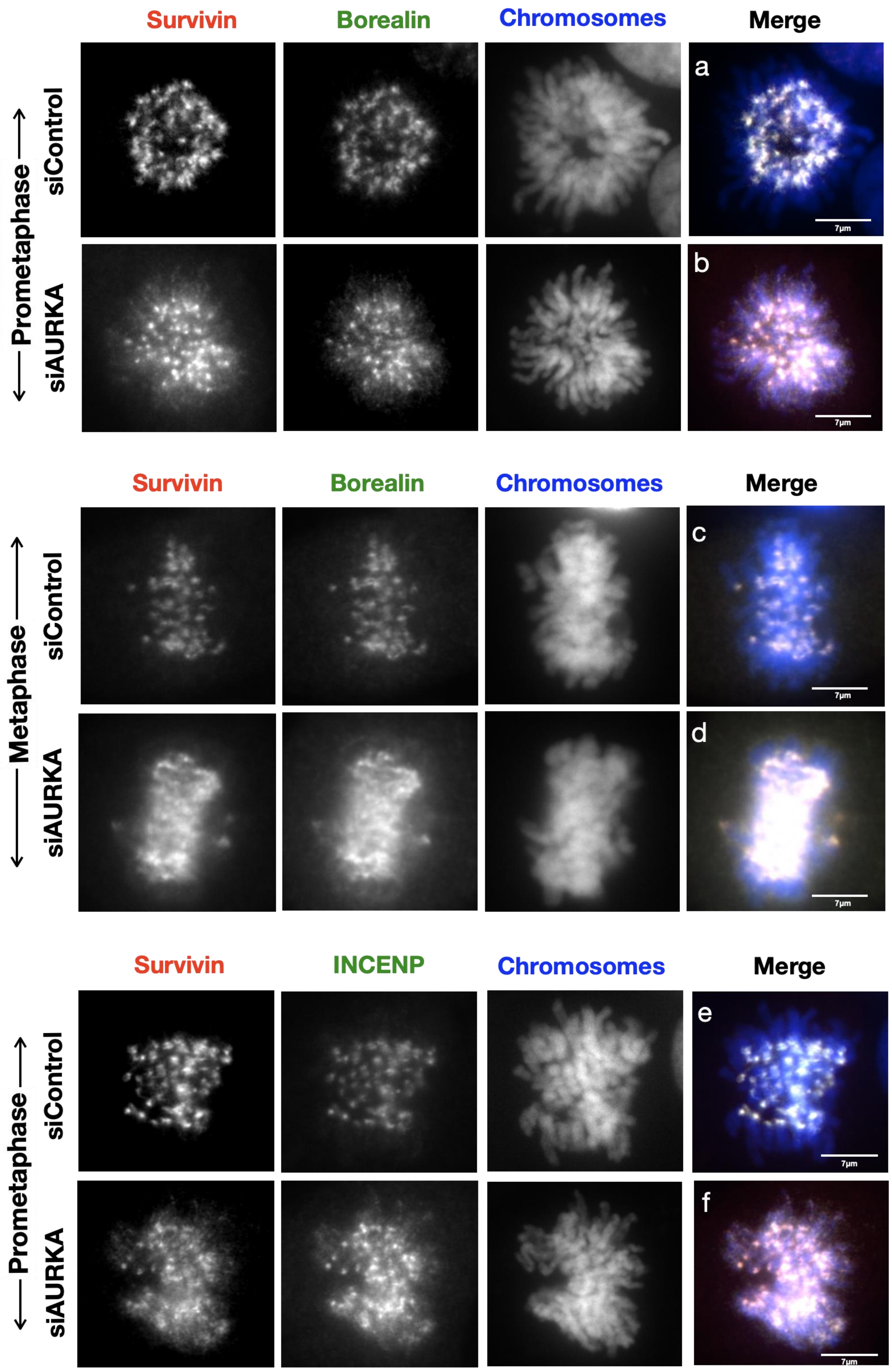

Fig. S7: Knocking down AURKA causes mislocalisation of CPC members INCENP and borealin during early mitosis.
A gallery of cells treated with either 40 nM scrambled-siRNA (siControl) or 40 nM AURKA-siRNA (siAURKA) for 48 h. Treated cells were fixed and immunostained for INCENP and borealin to examine their localisation during mitosis. Panels (a, b, c and d) immunostained for borealin (green), survivin (red) and chromosomes (blue) and panels (e and f) immunostained for INCENP (green), survivin (red) and chromosomes (blue). Scale bar: 7 µm. All the microscopy images shown are representative of three independent repeats.

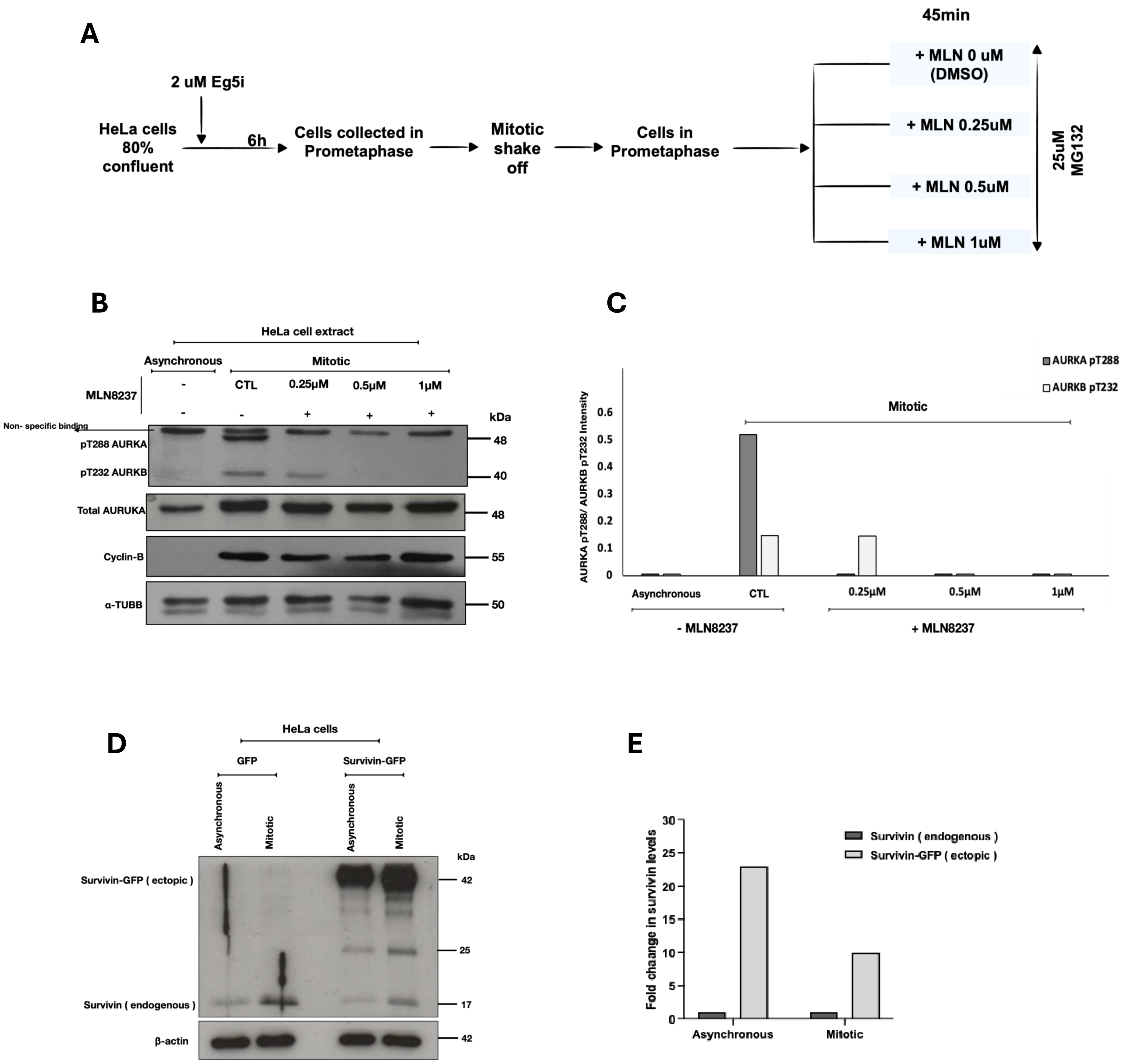

Fig. S8: Inhibiting AURKA phosphorylation using different concentrations of MLN and assessing survivin overexpression.
(A) Schematic demonstrating the method used for cell synchronisation and treatment. HeLa cells were arrested with 2 µM Eg5 inhibitor for 6 h and then treated with different MLN concentrations: 0.25 µM, 0.5 µM, and 1 µM for 45 min. **(B)** Western blot of asynchronous and mitotic HeLa cell lysate treated with control (DMSO) or with different concentrations of MLN, as described by S8A. The black arrow indicates non-specific bands. The cell lysate was immunoblotted against Thr288 AURKA and Thr232 AURKB to determine AURKA and AURKB activity, respectively. α-tubulin was used as a loading control, and cyclin B1 was used as a mitotic marker. **(C)** Graph represents the quantification of the intensity of the phosphorylation levels of both AURKA and AURKB, normalised to α-tubulin, represented by the western blot shown in (B). **(D)** Western blot comparing the levels of endogenous survivin and ectopically expressed survivin-GFP in asynchronous and mitotically arrested HeLa cells. β-actin was used as a loading control. **(E)** Graph showing the fold increase of survivin-GFP expression relative to endogenous survivin, based on the blot shown in (D). Quantification was normalised to β-actin. Data shown are from one representative experimental repeat.

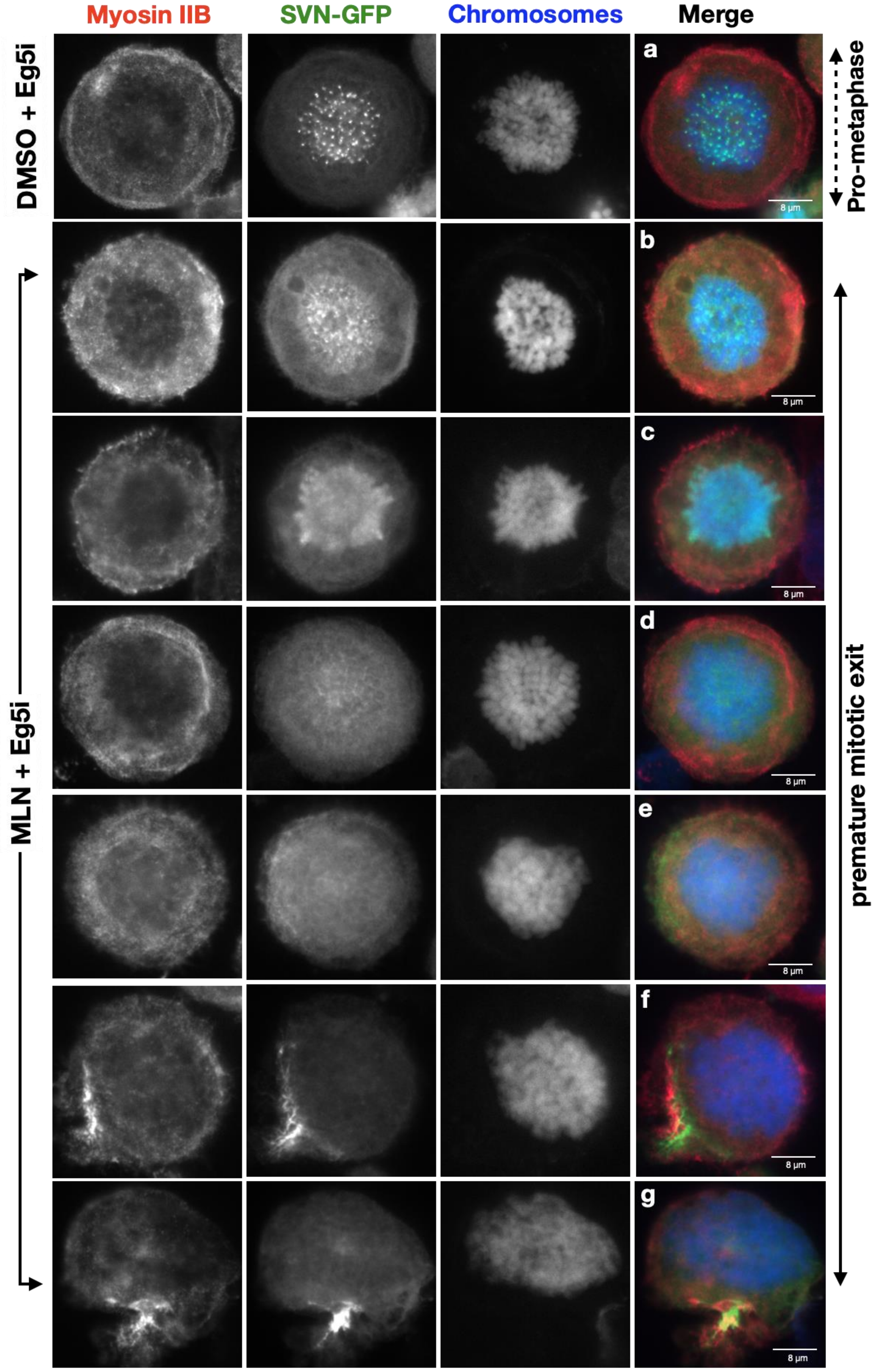

Fig. S9: Inhibiting AURKA activity caused cells to exit mitosis prematurely via forming an aberrant contractile ring.

A gallery of HeLa cells expressing survivin-GFP undergoing mitotic slippage. HeLa cells were arrested in prometaphase using a 2 µM Eg5 inhibitor, then treated with DMSO or 0.25 µM MLN, as illustrated in Fig. 4(A). Cells were fixed and immunostained for myosin IIB (red) and survivin-GFP (green) to examine their localisation throughout mitotic slippage. DAPI was used to stain the chromosomes (blue). Panel (a) represents a prometaphase-arrested cell treated with DMSO. Panels (b-g) cells treated with 0.25 µM MLN and undergoing different stages of premature mitotic exit. The images presented in this figure were obtained from HeLa cells stably expressing survivin-GFP following the experimental design shown in Fig. 4A. All the microscopy images shown are representative of three independent repeats.

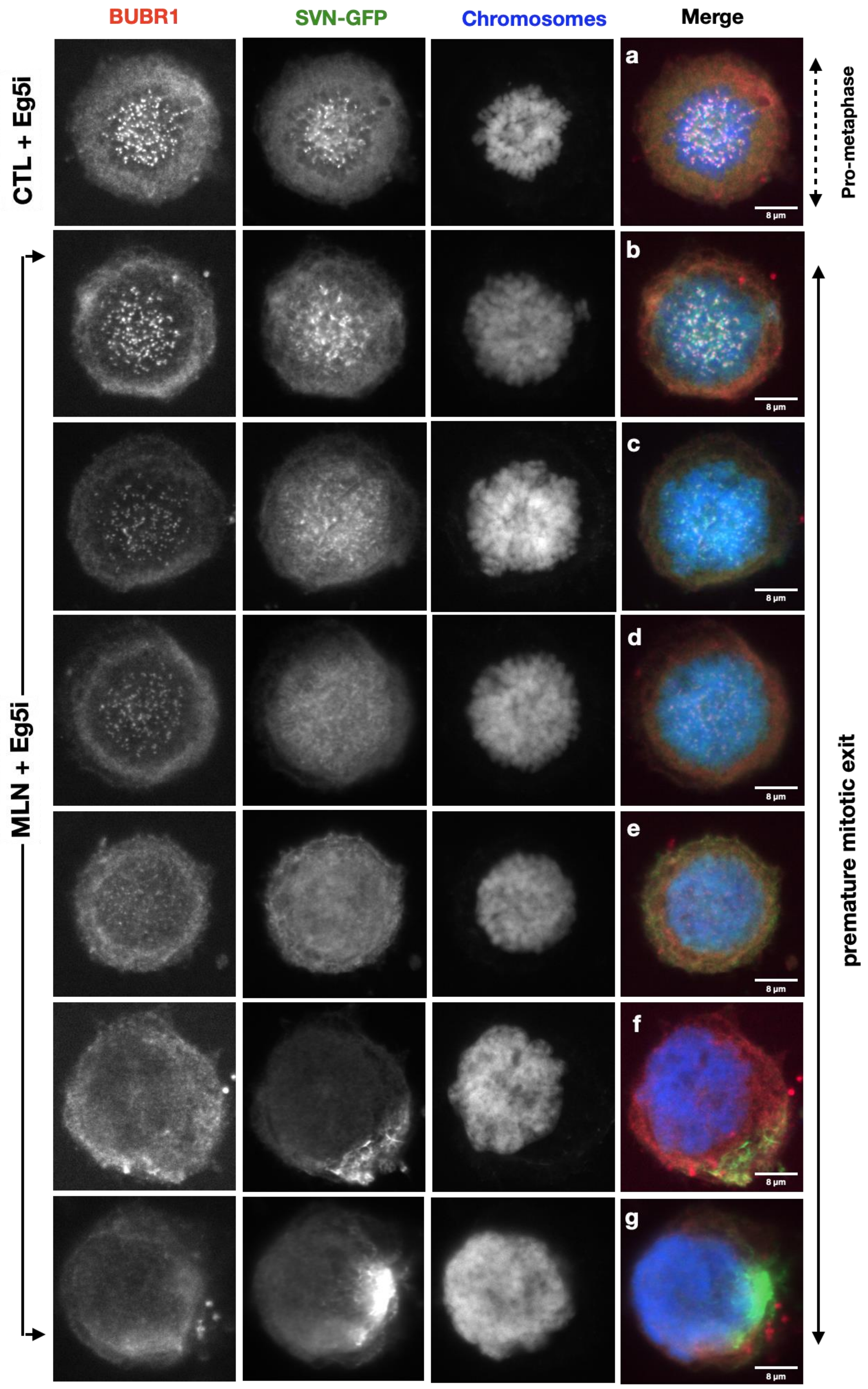

Fig. S10: AURKA inhibition causes a decrease in the levels of BubR1 at the unattached kinetochores.

A gallery of images showing stable HeLa cells expressing survivin–GFP at various stages of premature mitotic exit. The cells were arrested in prometaphase using an Eg5 inhibitor, then treated with DMSO or 0.25 µM MLN, as illustrated in Fig. 4(A). The cells were fixed and immunostained against BubR1 (red), chromosomes (Blue), survivin-GFP (green). Panel (a) represents a prometaphase-arrested cell treated with DMSO. Panels (b-g) represent cells treated with MLN and undergoing premature mitotic exit. Scale bar: 8 µm. The images presented in this figure were obtained from HeLa cells stably expressing survivin-GFP following the experimental design shown in Fig. 4A. All the microscopy images shown are representative of three independent repeats.

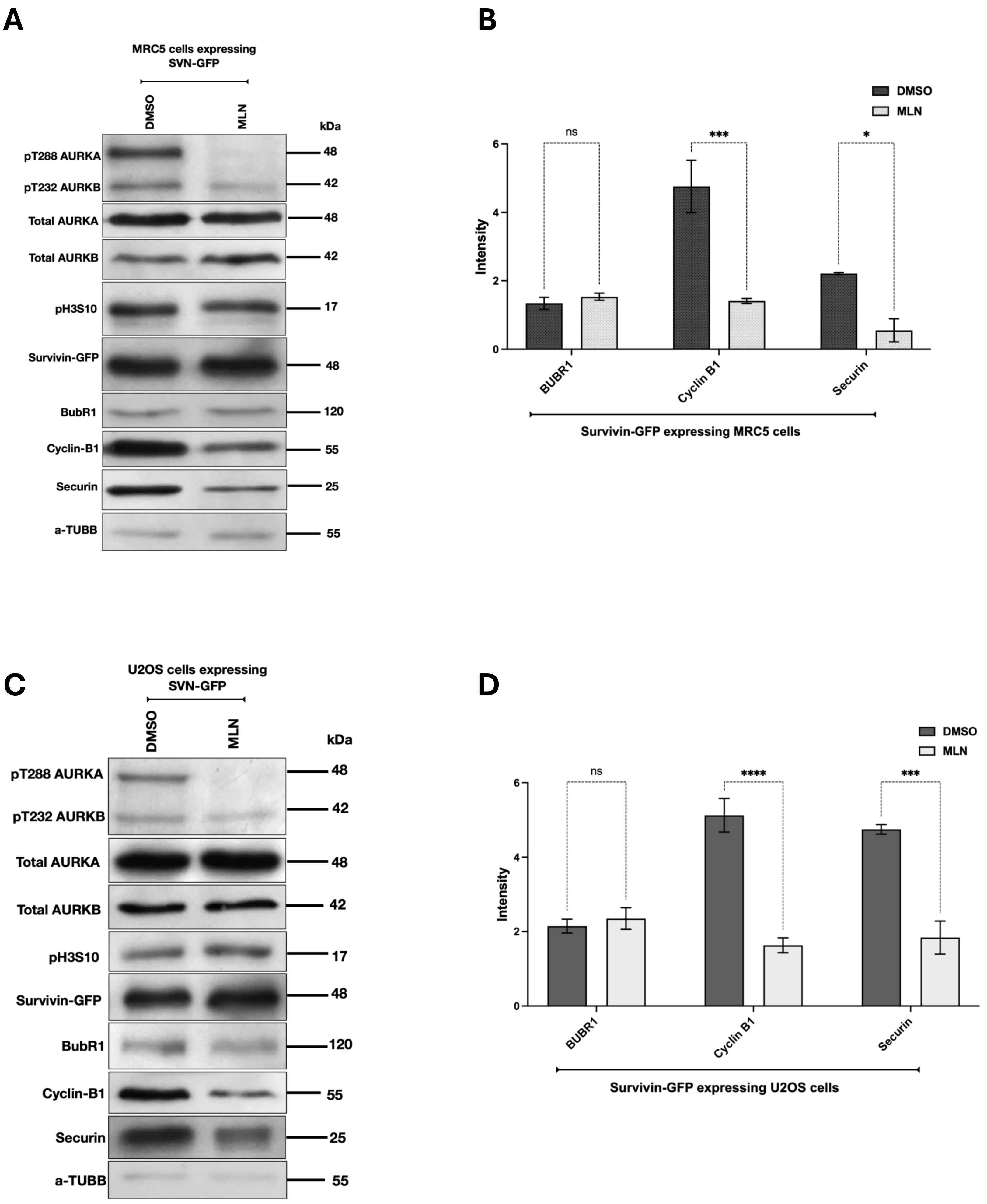

Fig. S11: AURKB inhibition enables cells with high levels of survivin to breach the SAC in MRC5 and U2OS cells. (A,C) Western blot of mitotically arrested stable MRC5 and U2OS cell lines expressing surviving-GFP treated with either DMSO or 1 μM MLN as illustrated in Fig 4. A. Cells were immunoblotted against BubR1, securin and cyclin B1. Thr288 AURKA and Thr232 AURKB were used to determine AURKA and AURKB activity, respectively. α-tubulin was used as a loading control. **(B,D)** Graphs demonstrate two-way ANOVA analysis of mean intensity of the expression levels of BubR1, cyclin B1 and securin, normalised to α-tubulin in stable MRC5 and U2OS cell lines expressing survivin-GFP and treated with either DMSO or 1 μM MLN. All results in this figure were obtained from MRC5 and U2OS cells stably expressing survivin-GFP following the experimental design shown in Fig. 4A. All blots shown in this figure are representative of two independent repeats. Quantitative data are presented as means ± SD (n = 2). *P* value (ns = non-significant, **P* < 0.05, ***P* < 0.01, ****P* < 0.001, **** *P* < 0.0001).
