# Supplementary material for: Survivin and Aurora Kinase A control cell fate decisions during mitosis
Source: Mol Oncol. 2025 Oct 12;20(3):727–52. doi: 10.1002/1878-0261.70141 (PMC13042919; doi:10.1002/1878-0261.70141)
Supplement: Supplementary file 2 — Table S1. Plasmid list. [file MOL2-20-727-s002.pdf]

Table S1: Plasmid list

Plasmid	Insert	Relevance	Cloning strategy/	Reference
pGEX4T1	SVN_{1-142}	full length SVN	EcoR1/Xho1, PCR	Colnaghi et al., 2010
pGEX4T1	SVN_{90-142}	C-terminal alpha helix		Colnaghi et al., 2010
pGEX4T1	SVN_{1-90}	BIR domain and N-terminus		Colnaghi et al., 2010
pGEX4T1	SVN_{20-90}	BIR domain	EcoR1/Xho1, PCR<br>Fwd primer:<br>Reverse primer:	This study
pGEX4T1	SVN_{1-10}	N-terminus (MTS)		Dunajová, et al., 2016
pGEX4T1	SVN_{1-30}	N-terminus		This study
pGEX4T1	SVN_{30-67}	Within BIR		This study
pGEX4T1	SVN_{60-90}	Within BIR		This study
pGEX4T1	SVN_{T34A}	Cdk1 site, not phosphorylatable	SDM	Barrett et al., 2009
pGEX4T1	SVN_{T34E}	Cdk1 site, phosphomimic	SDM	Barrett et al., 2009
pGEX4T1	SVN_{T48A}	CK2 site, not phosphorylatable	SDM	Barrett et al., 2011
pGEX4T1	SVN_{T48E}	CK2 site, phosphomimic	SDM	Barrett et al., 2011
pGEX4T1	$SVN_{DD70,71AA}$	Aurora-B binding site	SDM	This study
pcDNA3.1	SVN-GFP	Full length SVN tagged at C-terminus with GFP		Carvalho et al., 2003
pcDNA3.1	GFP			Carvalho et al., 2003
PVENUS-N1	AURKA			Dr. C. Lindon (Cambridge)
PBS	AURKB			Wheatley lab
